# Linking Metabolic Disorders and Immune System Phenomena in Schizophrenia: The Role of Adipose Tissue and Inflammation

**DOI:** 10.3390/biomedicines13092308

**Published:** 2025-09-20

**Authors:** Aleksandra Julia Oracz, Mateusz Zwierz, Maciej Naumowicz, Stefan Modzelewski, Maria Suprunowicz, Napoleon Waszkiewicz

**Affiliations:** 1 Department of Psychiatry, Medical University of Bialystok, pl. Wołodyjowskiego 2, 15-272 Białystok, Poland; stefan.modzelewski@sd.umb.edu.pl (S.M.); maria.suprunowicz@sd.umb.edu.pl (M.S.); napoleon.waszkiewicz@umb.edu.pl (N.W.); 2 Faculty of Medicine with the Division of Dentistry, Division of Medical Education in English, Medical University of Bialystok, Jana Kilińskiego 1, 15-089 Białystok, Poland; 39995@student.umb.edu.pl (M.Z.); 38741@student.umb.edu.pl (M.N.)

**Keywords:** obesity, schizophrenia, schizophrenia, cytokines, inflammation, adipose tissue, immune system phenomena, biomarkers, antipsychotic agents, metabolic disorders

## Abstract

Emerging evidence highlights the role of chronic low-grade inflammation and dysregulated cytokines in both obesity and schizophrenia, suggesting overlapping immune system pathways that contribute to cognition and nervous system inflammation. Excess adipose tissue functions as an active endocrine organ, releasing pro-inflammatory mediators that may serve as potential biomarkers, while the use of antipsychotic agents in schizophrenia further modifies cytokine profiles and immune responses. A key knowledge gap lies in understanding how adipose-related inflammation modifies the severity of psychotic symptoms, cognitive deficits, and the efficacy of antipsychotic medications. This review aims to present excess adipose tissue as a potential contributor to the development of SCZ or a modifier of treatment efficacy, emphasizing the role of immune imbalance, inflammatory pathways, and metabolic dysfunction. By synthesizing current findings, we aim to present obesity not only as a frequent comorbidity in schizophrenia but also as a potential driver of neuroinflammation and disease progression. Here, we demonstrate that excess adiposity may perpetuate a vicious cycle linking metabolic dysfunction, immune activation, and psychiatric symptomatology. Situating these findings within a broader context, the review underscores the clinical need for inflammation-informed, individualized management strategies that integrate psychiatric care with metabolic monitoring. Ultimately, clarifying the shared inflammatory pathways of obesity and schizophrenia may open new avenues for biomarker development and targeted interventions.

## 1. Introduction

Schizophrenia (SCZ) is a complex neuropsychiatric disorder with multifactorial etiology, affecting approximately 1% of the population worldwide [1]. Increasing evidence highlights the role of immune dysregulation and chronic low-grade inflammation as central processes shaping disease onset and course [2].

Within this broader framework, obesity emerges as a particularly important comorbidity of SCZ—not only as a side effect of pharmacological treatment, but also as a potential independent factor influencing the course of the disease [3]. Adipose tissue, especially visceral fat, has endocrine functions and serves as a source of numerous inflammatory mediators, its excess may intensify inflammatory processes already present in individuals with SCZ [4]. Epidemiological data indicate that the risk of developing obesity is up to twice as high in individuals diagnosed with SCZ (especially women) compared to the general population [5]. The presence of obesity in SCZ has been linked to more severe symptoms, greater cognitive deficits, and poorer treatment outcomes, although the mechanisms behind this relationship are not yet fully understood [6].

Pharmacological treatment contributes substantially to this problem. Antipsychotic medications, particularly second-generation antipsychotics (SGAs), are strongly associated with weight gain and metabolic dysfunctions [4,5]. For example, among patients with a first episode of schizophrenia (FES) previously untreated, 38.2% experienced ≥7% weight gain after just 8 weeks of antipsychotic therapy. Interestingly, weight gain was positively correlated with a decrease in Positive and Negative Syndrome Scale (PANSS) scores [7]. This finding is further supported by another study showing that initiation of treatment with second-generation antipsychotics (SGAs) resulted in an average 3.3% increase in body weight among patients with SCZ. Notably, nearly half of the participants were already obese at baseline [8]. The degree of weight gain depends on the specific antipsychotic used: although clozapine and olanzapine (both SGAs) are most frequently associated with weight gain, this side effect can occur with nearly any antipsychotic, regardless of generation, albeit to varying extents [9]. The lowest risk has been attributed to aripiprazole and ziprasidone [10]. Interestingly, some first-generation antipsychotics, such as molindone or pimozide, may even promote weight loss in the short term, although these findings remain inconclusive [11].

Yet, weight gain is not solely medication-related: even drug-naïve patients with SCZ may display higher visceral fat levels [12]. This suggests that poor hygiene and dietary habits, insufficient treatment of comorbid conditions and genetic predispositions also contribute [13]. Once again, this highlights SCZ as a complex disorder and a significant therapeutic challenge.

Taken together, these findings underscore the dual challenge faced by patients and clinicians: on one hand, effective pharmacological therapy is essential for symptom control; on the other, obesity and its inflammatory sequelae may aggravate psychiatric and somatic outcomes. Importantly, the interplay between SCZ, obesity, and inflammation has not been sufficiently integrated into current models of disease pathophysiology. The knowledge gap lies in understanding how adipose tissue-related inflammation modifies neuropsychiatric symptoms, cognitive decline, and treatment responsiveness in SCZ.

The present review addresses this gap by synthesizing current evidence on the role of obesity in shaping the immune-inflammatory background of SCZ. By framing obesity not only as a frequent comorbidity but also as a potential disease modifier, this work emphasizes the need for interdisciplinary approaches that integrate psychiatric care with metabolic monitoring. Early prediction of SCZ risk or symptom severity in individuals with excessive adipose tissue may allow for timely medical intervention and treatment, potentially reducing the risk of harmful behaviors and improving therapeutic outcomes.

## 2. Materials and Methods

As this work is a narrative review, no strict inclusion criteria were applied. The included studies were published between 2008 and 2025. Literature was searched via PubMed, Web of Science, and Google Scholar between 2008 and 2025 using relevant keywords such as: “obesity, schizophrenia, inflammatory theory of schizophrenia, Th1/Th2 imbalance, metabolic biomarkers of schizophrenia”. Both original research articles and review papers were considered during initial selection. Zotero software (version 7.0.15, 64-bit) was used to remove duplicates. Conference abstracts and non-English publications were excluded based on title and abstract screening.

To provide a comprehensive overview of the inflammatory markers associated with SCZ, we focused on studies reporting levels or roles of specific cytokines. The search was conducted in PubMed using specific terms for each cytokine. The number of screened and included original articles is summarized in Table 1.

In addition, for the section “Potential Cross-Talk Between Obesity-Related Inflammation and Schizophrenia”, a focused search was conducted using combinations of terms such as “obesity schizophrenia inflammation”, “adipose tissue cytokines schizophrenia”, and “metabolic inflammation schizophrenia”. Based on this search, 10 original articles published between 2008 and 2025 were included.

While this review aims for a broad perspective, it is not systematic and has notable limitations. Nonetheless, the selection process was structured to ensure a representative synthesis of the current literature.

## 3. Obesity-Induced Inflammation and Schizophrenia Symptoms

**Figure 1:**
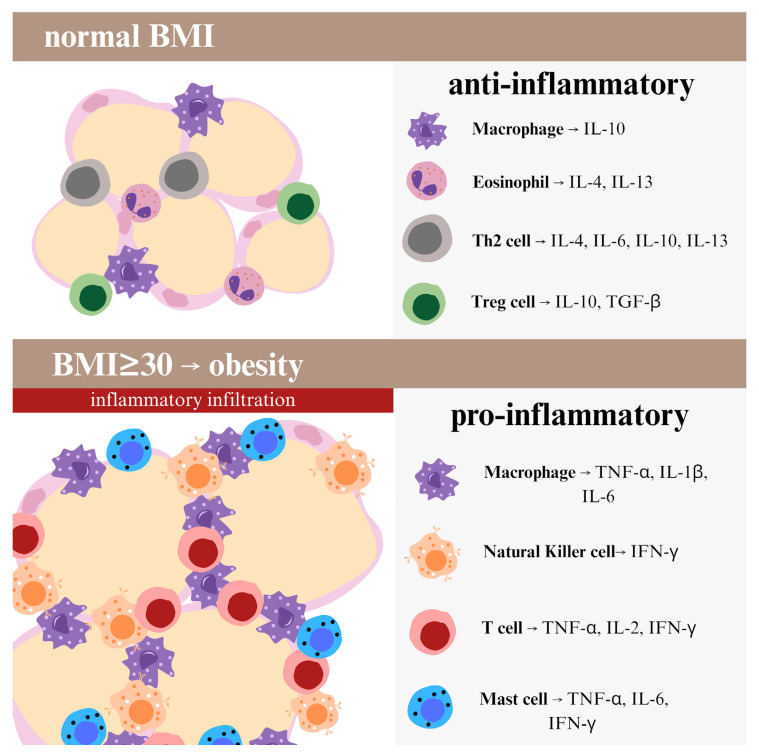
The figure shows differences in the composition of immune cells and pro- and anti-inflammatory cytokine profiles in a person with normal body fat content and in an obese person. Normal adipose tissue is characterized by an anti-inflammatory cytokine composition. Macrophages produce interleukin-10 (IL-10) and eosinophils—interleukin-4 (IL-4) and interleukin-13 (IL-13). Type 2 T helper cells produce IL-4, interleukin-6 (IL-6), IL-10 and IL-13, while regulatory T cells (Treg) secrete IL-10 and transforming growth factor beta (TGF-β). The adipose tissue of obese individuals has a pro-inflammatory cytokine profile. Macrophages produce tumor necrosis factor α (TNF-α), interleukin-1 β (IL-1β) and IL-6. Natural killer cells release interferon-γ (IFN-γ), while T cells produce TNF-α, interleukin-2 (IL-2) and IFN-γ. Mast cells are a source of TNF-α, IL-6 and IFN-γ.

Obesity is a well-documented condition associated with low-grade chronic inflammation, which may be relevant in the context of mood disorders [14]. This association is well known and thoroughly documented. Excessive adipose tissue functions as an active endocrine organ that secretes pro-inflammatory cytokines and adipokines such as leptin. With adipocyte hypertrophy and macrophage infiltration, leptin levels increase, promoting a shift toward a pro-inflammatory Th1 profile and reducing Th2-related anti-inflammatory activity [15]. This immune imbalance fosters a state of chronic low-grade inflammation that may be a key mechanism linking obesity and mood disorders (Figure 1).

Consistent with this immunometabolic background, higher body mass index (BMI) has been associated with reduced total gray matter and smaller volumes of the bilateral orbitofrontal, prefrontal, and broader frontal cortices—changes that may be associated with more severe residual symptoms in patients with SCZ [16]. It can be suggested that although inflammation is not a predictor of suicide attempts in SCZ, it is associated with the degree of suicide risk in this disorder [17].

Inflammatory mediators such as interleukin-6 (IL-6), tumor necrosis factor-alpha (TNF-α), and C-reactive protein (CRP) are thought to affect the nervous system through activation of the kynurenine pathway, leading to a decrease in serotonin levels and an increase in neurotoxic metabolites such as quinolinic acid—a known N-Methyl-D-Aspartate (NMDA) receptor agonist. This, in turn, promotes excitotoxicity, intensifies neuroinflammation, and contributes to depressive symptoms, anhedonia, and impulsivity, which are frequently observed in SCZ, particularly during its acute phases.

Moreover, these cytokines impair the functioning of the hypothalamic–pituitary–adrenal (HPA) axis, increasing vulnerability to stress and mood disturbances. Therefore, it has been hypothesized that anti-inflammatory interventions may potentially reduce suicidal tendencies [18].

It is also suggested that disruptions in cytokines such as IL-6 and interleukin-10 (IL-10) may contribute to hippocampal volume reduction—a region crucial for cognitive and emotional processing [19]. Animal studies have shown elevated IL-6 mRNA levels in the hippocampus in induced models of SCZ [20], and in humans, a correlation was observed between IL-6 promoter methylation in peripheral leukocytes and the integrity of the corpus callosum, a structure responsible for interhemispheric coordination [21]. Taken together, these findings suggest that obesity-related inflammation may contribute not only to structural brain alterations but also to the variability in clinical symptom severity observed in individuals with SCZ.

## 4. Imbalance of the Th1/Th2 Axis in Schizophrenia

Pro-inflammatory cytokines are key mediators of the immune response, playing a critical role in regulating inflammatory processes and modulating central nervous system (CNS) function. In the context of SCZ, an increasing number of studies point to immune dysfunction characterized by an imbalance between pro- and anti-inflammatory cytokines [22,23,24]. Some studies indicate a dominance of T helper type 1 (Th1) responses [25], while other findings suggest a predominance of T helper type 2 (Th2) cytokines, particularly in patients experiencing a first episode of psychosis (FEP) [26]—especially those with concomitant depressive symptoms [27]—or during acute relapses [26]. Of particular note are the findings of Borovčanin et al., who reported a Th2 predominance in the combined cohort of psychotic patients (FEP and relapse) as indicated by elevated IL-4 levels. However, after separating the groups, this predominance was mainly evident in patients with SCZ in relapse, compared to both FEP and healthy controls [26].

**Figure 2:**
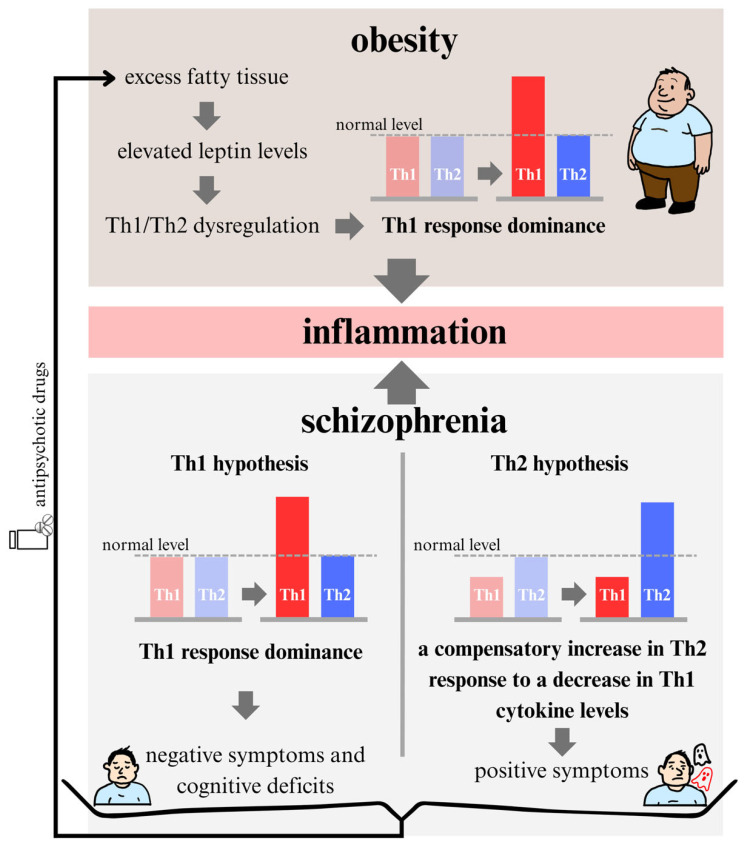
The graphic illustrates chronic inflammation as a shared pathophysiological mechanism linking obesity and schizophrenia (SCZ) through Th1/Th2 immune imbalance. In obesity, elevated leptin levels (driven by increased adipose tissue mass) promote Th1 dominance and contribute to a chronic pro-inflammatory state. In SCZ, two competing immunological hypotheses are proposed: the first posits Th1 predominance, associated with negative symptoms and cognitive deficits; the second suggests a primary reduction in Th1-related activity followed by a compensatory increase in Th2 responses, which may underlie the emergence of psychotic symptoms.

The inconsistency of the imbalance between pro- and anti-inflammatory cytokines may reflect the dynamic nature of the Th1/Th2 axis, which is influenced by the stage of illness, patients’ metabolic status (including obesity), pharmacological treatment, and environmental factors. One hypothesis proposes that the immune imbalance in SCZ is characterized by a primary reduction in Th1-associated cytokines, accompanied by a compensatory increase in Th2 cytokines. This pattern suggests that Th1 suppression may be the initiating event, while Th2 elevation constitutes an attempted, but ultimately maladaptive, compensatory response. Instead of restoring immunological balance, this shift reflects a pathological inflammatory process that plays a central role in the pathophysiology of SCZ [28] (Figure 2). Notably, the Th1/Th2 imbalance described above appears to be specific to SCZ. Although a disruption of Th1/Th2 balance is also observed in obesity, the underlying mechanism differs. In obesity, inflammation is primarily mediated by innate immune activation, metabolic stress, and dietary factors, such as antioxidant intake [29]. Therefore, despite both conditions involving systemic inflammation, their immune mechanisms differ.

### 4.1. The Role of Pro-Inflammatory Interleukins in the Pathophysiology of Schizophrenia

#### 4.1.1. IL-6

In the context of pro-inflammatory cytokines, researchers most frequently focus on the role of IL-6, whose elevated levels have been associated with SCZ, FEP, and clinical high-risk states for psychosis [30]. It has been suggested that IL-6 mRNA are elevated in brain tissue of individuals with SCZ [31], while peripheral IL-6 levels are consistently increased regardless of disease phase [32], potentially contributing to impaired corpus callosum integrity [33] and disrupted neuroplasticity via reduced brain-derived neurotrophic factor (BDNF) expression [34]. Moreover, the impact of IL-6 on neuronal plasticity and neurotransmission pathways has been suggested to underlie the development of psychotic symptoms [23] and may also be linked to their exacerbation [35].

The previously mentioned observation on altered BDNF expression is particularly salient in the context of the development of obesity as a complication of antipsychotic treatment, as research has demonstrated a correlation between BMI increase and a gradual decline in BDNF levels as obesity progresses. Elevated BDNF levels have been shown to promote satiety, while BDNF deficiency has been associated with impaired satiety, a factor that can contribute to the development of obesity. The effect is bidirectional: obesity has been shown to reduce BDNF levels, and SCZ, by increasing IL-6 levels, also contributes to its reduction. These alterations have been shown to exacerbate each other, thereby creating a vicious cycle that leads to the exacerbation of both psychiatric and metabolic symptoms, and consequently complicates patient treatment [36].

In SCZ patients, higher IL-6 levels have been associated with more severe depressive symptoms [37], cognitive dysfunction [34], negative symptoms [37], and self-deprecation [38]. Elevated IL-6 may also contribute to reduced affect and alogia (possibly through modulation of TNF-α) [39], as well as NMDA receptor dysfunction, which can exacerbate positive symptoms [40]. Glutamatergic neurotransmission and NMDA receptor-associated neuroplasticity are essential for maintaining normal body weight. In obesity, NMDA dysfunction impairs spatial learning and memory, highlighting its dual role in both psychiatric symptoms and metabolic dysregulation [41]. Findings suggest that the relationship between IL-6 and symptom severity may vary depending on the stage of the illness (e.g., in early-onset schizophrenia (EOS), IL-6 may be neurotoxic, while in adult-onset schizophrenia (AOS), it may have a neuroprotective role), warranting cautious interpretation. Due to the immunomodulatory effects of antipsychotic medications on IL-6 levels [42], including patients undergoing pharmacological treatment in studies, results in conflicting findings regarding the aforementioned correlations [37,43,44,45,46]. However, it is crucial to consider that chronically elevated IL-6 levels, common in both SCZ and obesity, may create a vicious cycle, with each condition exacerbating the other.

Notably, SGAs such as risperidone, olanzapine, aripiprazole, and quetiapine have been shown to reduce IL-6 levels, which have been correlated with improved cognitive functions (e.g., working memory, verbal learning) [47], similar to findings with escitalopram, where IL-6 reduction was associated with decreased severity of negative and cognitive symptoms on the PANSS [48]. Conversely, Li et al. demonstrated that olanzapine significantly raises IL-6 levels, which correlates with increased insulin resistance (IR) and promotes obesity development through inflammatory mechanisms. The elevated IL-6 levels associated with olanzapine use not only indicate neuroinflammation but also suggest an exacerbation of inflammation in adipose tissue, which may promote fat accumulation. Similar observations regarding olanzapine were reported by Calevro et al., whereas haloperidol did not induce a significant increase in pro-inflammatory IL-6 production in adipose tissue [49,50].

#### 4.1.2. TNF-α

Another pro-inflammatory cytokine with significant relevance to the pathophysiology of SCZ is TNF-α, previously mentioned in this paper. While some studies have reported elevated TNF-α levels in patients with SCZ [22,51,52], including those experiencing a first episode [53,54], a growing body of evidence suggests that increased peripheral TNF-α may also serve as a potential biomarker of remission, reflecting systemic immune modulation associated with symptom stabilization [55,56,57,58]. The lack of statistically significant differences or reduced TNF-α levels observed in some studies [59,60,61] is often attributed to an uneven sex distribution, given that TNF-α levels are generally lower in men [62]. This may mean that the TNF-α-mediated inflammatory response may be stronger in women with SCZ and comorbid obesity, as women tend to have a higher percentage of body fat, further exacerbating both metabolic and psychiatric symptoms.

Most studies involving patients with SCZ do not report significant correlations between TNF-α levels and cognitive performance [53,57], overall functioning, or positive and negative PANSS scores [60,61]. However, some studies have suggested that elevated TNF-α levels may contribute to deficits in working memory [63,64].

In patients treated with paliperidone (both alone and combined with sertraline) [65], or with olanzapine over 6 weeks [60], TNF-α levels increased after treatment, higher levels were negatively correlated with negative symptoms, general psychopathology, and total PANSS scores [66], possibly reflecting an adaptive immune response modulated by long-term antipsychotic treatment. In contrast, aripiprazole exerts an opposite effect—it lowers TNF-α and leptin levels and positively influences body weight, BMI, and percent body fat (BF%) [67].

#### 4.1.3. CRP

Numerous studies have reported elevated levels of CRP across different phases of SCZ, suggesting its potential as a useful biomarker for diagnosing and monitoring the course of the disorder Moreover, serum CRP reflects systemic inflammation, making it one of the most widely used markers of systemic inflammatory status [68]. It should be noted that CRP may contribute to disruptions in the integrity of the blood–brain barrier, increasing its permeability to inflammatory mediators and cytokines, thereby promoting neuroinflammation [69]. Higher high-sensitivity C-reactive protein (hs-CRP) levels in the SCZ group were associated with female gender, more severe negative symptoms, a greater number of comorbidities, and worse metabolic risk factors, including BMI [70].

One study demonstrated that CRP levels significantly increase during acute psychotic episodes and may decrease after resolution of the acute phase [71]. Another study reported that patients with chronic SCZ exhibit elevated inflammatory status, whereas those with recent-onset disease show lower levels [72].

In a study focused on the acute phase of SCZ, significantly elevated serum CRP concentrations were observed. Importantly, CRP levels were positively correlated with the severity of typical clinical symptoms, suggesting a potential link between inflammatory activity and the intensity of psychopathological manifestations [73].

It has been documented that higher CRP levels in patients with SCZ are mainly associated with more severe negative symptoms, increased aggression, and agitation [74,75,76]. Additionally, studies have shown that elevated CRP in SCZ is linked to impairments in various cognitive functions, including working memory and learning ability [77]. Another study found a significant association between elevated CRP levels and a history of suicide attempts in individuals with SCZ. CRP levels at the 75th percentile were associated with a 2.6-fold increased risk of a suicide attempt, while levels at the 90th percentile corresponded to a 3.3-fold increase [78].

Elevated CRP may also serve as a biomarker indicating a poorer clinical course of schizophrenia during the first year after diagnosis [79], as well as an increased risk of suicide [78]. Furthermore, longitudinal studies have shown a correlation between CRP levels during adolescence and the later diagnosis of SCZ in adulthood [80].

CRP is a promising biomarker in psychiatry, also for practical reasons, as it is relatively stable, easy to measure, and consistently assessed in most diagnostic laboratories.

#### 4.1.4. IL-1β

In the context of inflammatory hypotheses in SCZ, particular attention has been paid to abnormal microglial activation and dysregulated interleukin-1 beta (IL-1β) signaling. Multiple studies have reported elevated IL-1β mRNA expression in microglia from individuals with SCZ [81,82], particularly in its chronic form, relative to healthy controls [83]. The observed positive correlation between IL-1β levels and illness duration underscores the specific involvement of this cytokine in the pathophysiology of chronic SCZ [84].

These observations have been consistently replicated in other studies, which also report elevated plasma IL-1β levels in SCZ patients, including those experiencing a FES [43,53], suggesting that IL-1β-mediated inflammatory processes are already active at the early stage of psychosis [34,85]. In SCZ patients, IL-1β levels were associated with increased severity of positive symptoms and overall psychopathology on the PANSS [85], as well as with cognitive deficits (attention, working memory, sustained attention, and social cognition [86]) and impaired executive functions, similarly to IL-6 [34].

These associations may be further exacerbated by comorbid metabolic dysfunction (MD), which appears to interact with IL-1β signaling. In chronic SCZ patients, it was observed that men with MD had higher IL-1β levels than men without MD, while no significant association between MD and IL-1β levels was found in women. Furthermore, the analysis showed that among SCZ patients with MD, men had higher scores on the positive symptom scale and higher IL-1β levels than women with SCZ and MD, and then men with SCZ but without MD [87].

Long-term studies did not observe significant changes in IL-1β levels after 12 months of antipsychotic treatment (aripiprazole, olanzapine), regardless of sex. Similar results were found for IFN-γ and IL-10 [88]. Other analyses showed that a four-week therapy with atypical antipsychotics also did not normalize elevated levels of this cytokine, indicating its relative resistance to short-term pharmacological intervention [53].

#### 4.1.5. IL-2

Although available meta-analyses have found no evidence of differences in interleukin-2 (IL-2) levels between FES patients, those with acute exacerbations, and healthy controls [89,90], newer studies published after these analyses continue to show that IL-2 tends to be elevated in patients with SCZ, particularly during the FEP [23,91,92,93], acute exacerbation [62], and in those with chronic forms of the disorder [94,95]. This may be partly explained by IL-2 gene polymorphisms, such as the GG genotype, which could increase the risk of developing SCZ [96]. Furthermore, no association has been found between illness duration and IL-2 levels [97].

Nevertheless, some studies reported that higher IL-2 levels were associated with greater severity of positive and negative symptoms, general psychopathology, and higher total PANSS scores [60]. Similarly, in patients with treatment-resistant schizophrenia (TRS), IL-2 levels positively correlated with total PANSS scores. Interestingly, these correlations between pro-inflammatory cytokine levels and PANSS scores were not observed in TRS patients with comorbid metabolic syndrome (MetS) [98]. Interestingly, in contrast to the IL-1β findings, this unexpected result may suggest that metabolic dysfunction has an opposing effect on IL-2, potentially contributing to a decrease in IL-2 levels. However, further detailed studies are required to elucidate the mechanisms underlying this relationship. Recent studies have indicated a correlation between elevated BMI and reduced IL-2 levels, as well as attenuated negative symptoms and an unfavorable lipid profile in individuals diagnosed with chronic SCZ [99]. IL-2 has been considered a potential biomarker of impaired real-world functioning in SCZ. It has also been proposed as a biomarker of negative symptoms, such as amotivation and anhedonia, in people with SCZ, unlike IL-1β, which appears to be a marker of more general symptoms [100]. Notably, IL-2 levels did not significantly affect psychomotor performance, indicating a more selective association with motivational rather than motor domains of functioning [101].

A longitudinal analysis found no statistically significant changes in IL-2 levels over 52 weeks of antipsychotic treatment (aripiprazole, olanzapine) in either male or female patients with SCZ or SCZ spectrum disorders [88]. In contrast, another study showed that short-term treatment reduced IL-2 levels, but after 5 years, levels increased again despite symptom improvement—suggesting that the anti-inflammatory effects of antipsychotics may be temporary, with possible secondary immune activation over time [92]. Similarly, short-term treatment (4–10 weeks) with risperidone, olanzapine, or haloperidol lowered IL-2 levels, whereas no such effect was observed following clozapine treatment over the same period [90].

#### 4.1.6. IFN-γ

Literature data suggest that interferon gamma (IFN-γ) may play a distinct role in the pathophysiology of SCZ by linking peripheral immune activation with central glutamatergic [102] and dopaminergic dysfunction (via enhanced STAT1 signaling) [103]. This assertion is particularly salient in the context of obesity, as both glutamatergic and dopaminergic pathways, in conjunction with STAT1, play a pivotal role in the regulation of appetite and food intake. Dysregulation of these pathways has been demonstrated to contribute to the metabolic disturbances that are commonly observed in individuals with obesity.

Despite numerous studies, findings regarding serum IFN-γ levels in SCZ remain inconsistent – some reports indicate elevated levels [62,104] (FES and ultra-high risk (UHR) for psychosis patients [105]), increased IFN-γ mRNA expression [106], and higher IFN-γ concentrations in cerebrospinal fluid (CSF) [107], while others suggest decreases [108,109,110], and still others show no significant differences compared to controls [43,59,111,112,113], depending on disease phase and methodological differences.

IFN-γ shows broad relevance to SCZ, being linked to both positive symptoms and affective dimensions [114]. Notably, its link with psychomotor speed in SCZ patients appears particularly strong compared to other symptom domains [101].

Logistic regression analysis aimed at predicting treatment response showed no significant association between IFN-γ levels and antipsychotic response in patients with stabilized psychotic disorders [115]. Several studies have indicated that several weeks of treatment with SGAs—including risperidone, olanzapine, and aripiprazole—may reduce IFN-γ levels in patients with SCZ, particularly during FES and acute exacerbations [97,116]. This decrease has been associated with improvements in negative symptoms and overall functioning [116], although these effects appear to be short-term and less consistent with prolonged treatment [93]. In contrast, no changes in IFN-γ levels were observed with long-term antipsychotic treatment (12 months) using amisulpride, aripiprazole, and olanzapine [88].

#### 4.1.7. IL-17

Numerous studies have shown that interleukin-17 (IL-17) levels are elevated in patients with FES [117] and chronic SCZ [118], indicating that IL-17 plays a key role in the early stages of SCZ development and sustains the activation of the inflammatory axis throughout the disease [119]. However, its diagnostic and prognostic value has been questioned due to conflicting results [120,121].

No correlation was found between IL-17 levels and negative symptoms (PANSS-N) [122], and the positive correlation with positive symptoms and total PANSS scores is a rare and less reliable finding [123]. A consistent observation, however, is the correlation between IL-17 levels and cognitive symptoms (potentially due to its influence on lipid metabolism disturbances) [124], particularly in visuospatial/executive and language domains [125] (Table 2). Obesity, which frequently accompanies SCZ, predisposes to dyslipidemia and elevated triglyceride levels [126]. Diets rich in triglycerides further aggravate these disturbances, leading to stronger activation of inflammatory pathways, including the IL-17 signaling cascade [124]. Since IL-17 has been linked to cognitive impairment, this metabolic–inflammatory interaction may explain why patients with SCZ are particularly vulnerable to triglyceride-induced cognitive decline. Experimental studies support this by showing that high-triglyceride diets can exacerbate obesity-related cognitive impairment, reduce BDNF levels, impair cellular proliferation, and promote hippocampal apoptosis [127]. Thus, dietary and metabolic factors may synergize with IL-17–mediated inflammation, amplifying cognitive dysfunction in SCZ.

Similarly to earlier findings, differences are observed depending on the duration of therapy. In the case of short-term treatment, such as a 10-week SGA monotherapy (e.g., risperidone), although levels of IL-2, interleukin-4 (IL-4), IL-6, interleukin-8 (IL-8), IL-17, TNF-α, and IFN-γ tended to decrease following SGA monotherapy, the reduction in IL-17 was not statistically significant [97,118]. However, after 6 months of therapy, a significant decrease in IL-17 was observed, suggesting that this cytokine might serve as a biomarker of the acute SCZ phase, normalizing with long-term pharmacotherapy [116].

### 4.2. The Role of Anti-Inflammatory Interleukins in the Pathophysiology of Schizophrenia

#### 4.2.1. IL-4

There are highly conflicting reports in the literature regarding the anti-inflammatory role of IL-4 in SCZ, with some studies indicating a decrease in levels [143] while others reported no alteration (FES [34,60,105], stabilized SCZ [144], acute SCZ in CSF [112] and plasma [59], exacerbation SCZ [145]) or an increase (FES [92,93,111]), which may seem counterintuitive in the context of the dominant hypothesis assuming a pro-inflammatory nature of the disorder [91].

Higher IL-4 levels in SCZ patients have been associated with a reduction in the severity of symptoms such as psychosis, hostility, agitation, and mannerisms [133]. However, the correlation with negative symptoms remains ambiguous [60,134,146]. Increased IL-4 levels have been associated with enhanced language performance [133] and cognitive function in patients with SCZ [136], with no observed association with psychomotor speed [101]. Elevated IL-4 levels may also exert a protective effect against the development of psychotic symptoms and be associated with enhanced emotion recognition [137]. In early SCZ patients, especially those with neurological symptoms, IL-4 distinguished them from healthy controls, suggesting a link to neurodevelopmental mechanisms [147].

Both short-term (4-week) treatment [91] with risperidone and long-term (5-year) treatment failed to reduce IL-4 levels [92]. Interestingly, a reduction in IL-4 was observed after one year of treatment, and this alteration was specific to SCZ patients with predominantly depressive symptoms [148]. In addition, when examining the effect of gender on IL-4 levels, we found that although women had lower IL-4 levels at the beginning of the study compared to men, after 52 weeks of treatment, there was a noticeable increase in IL-4 levels in women, while a contrary effect was noted in men [88]. This underscores the importance of considering gender differences in future studies, as they may affect efficacy and immune responses to antipsychotic treatment. This change may also be related to differences in fat tissue proportions between genders. The reduction in IL-4 levels after treatment mirrors the pattern seen in obesity, where individuals with obesity and IR exhibit significantly lower IL-4 levels in both adipose tissue and plasma compared to lean individuals. IL-4 plays a crucial role in fat tissue metabolism and leptin secretion regulation, further linking immune system dysregulation to metabolic disturbances in obesity [149].

#### 4.2.2. IL-10

The literature on IL-10 in SCZ is highly inconsistent. While some studies report reduced IL-10 concentrations in SCZ [133], others indicate increased levels [38,49,54,119], especially at early stages of the illness [147,150], or find no significant changes at all [34,62,139,151,152,153]. Interestingly, a reduction in IL-10 was associated with a higher risk of developing a chronic disease course [133]. Higher IL-10 levels were present in SCZ patients with more severe depressive [152] and anxiety symptoms [154], and higher IL-10 levels were also associated with lower global functioning (GAF scale) [151], as well as impaired (slower) performance on psychomotor tests [153].

The results regarding the correlation between IL-10 and PANSS subscales are inconsistent [60,115,123,133,139,140,146], likely due to a suggested nonlinear or threshold effect, evident when PANSS scores exceed 85 [123]. Genetic variants have been demonstrated to intensify symptoms of avolition, apathy [155], cognitive performance [156,157,158], and dyskinesia [159]. Studies suggest that these cytokines which is produced by metabolically active visceral adipose tissue can cross the blood–brain barrier, affect the brain’s immune system and induce the expression of pro-inflammatory genes. Interestingly, increasing BMI is associated with a significant decrease in IL-10 mRNA expression in the human frontal cortex, which may affect cognitive function, brain adaptability and increase brain sensitivity to noxious stimuli [160].

Variability in IL-10 levels may be explained by antipsychotic pharmacotherapy. A significant positive correlation has been found between antipsychotic dose (expressed as chlorpromazine equivalent) and serum IL-10 concentration [138]. Similarly, SCZ patients treated with olanzapine or clozapine also exhibited higher IL-10 levels, with further increases observed after 6 weeks of olanzapine therapy [60,161]. These findings suggest that IL-10 levels are influenced by the type and duration of antipsychotic treatment.

#### 4.2.3. IL-13

Existing studies rarely include interleukin-13 (IL-13) in the context of SCZ, leaving the role of this cytokine in the disease’s pathophysiology full of questions and requiring further, more detailed analyses. No changes in IL-13 mRNA expression were observed in the prefrontal cortex [162]; however, in patients with FES, peripheral IL-13 levels were higher [163,164]. Despite this, no significant correlations were found between IL-13 levels and the severity of SCZ symptoms assessed by the PANSS [115]. However, IL-13 levels have been associated with cognitive domains, independently of the severity of classical psychotic symptoms [133].

Although IL-13 is classified as an anti-inflammatory Th2 cytokine, authors have noted that within the CNS, it may exert pro-inflammatory functions, enhancing the neurotoxic effects of reactive oxygen species (ROS) [164]. Despite higher IL-13 levels in SCZ patients [133], research findings did not fully confirm the theory of a shift toward Th2 lymphocytes in SCZ [121]. Nevertheless, IL-13 has been found to have high sensitivity and moderate specificity in predicting SCZ progression [163]. Moreover, no statistically significant association was observed with the response to antipsychotic treatment [115].

#### 4.2.4. TGF-β

**Figure 3:**
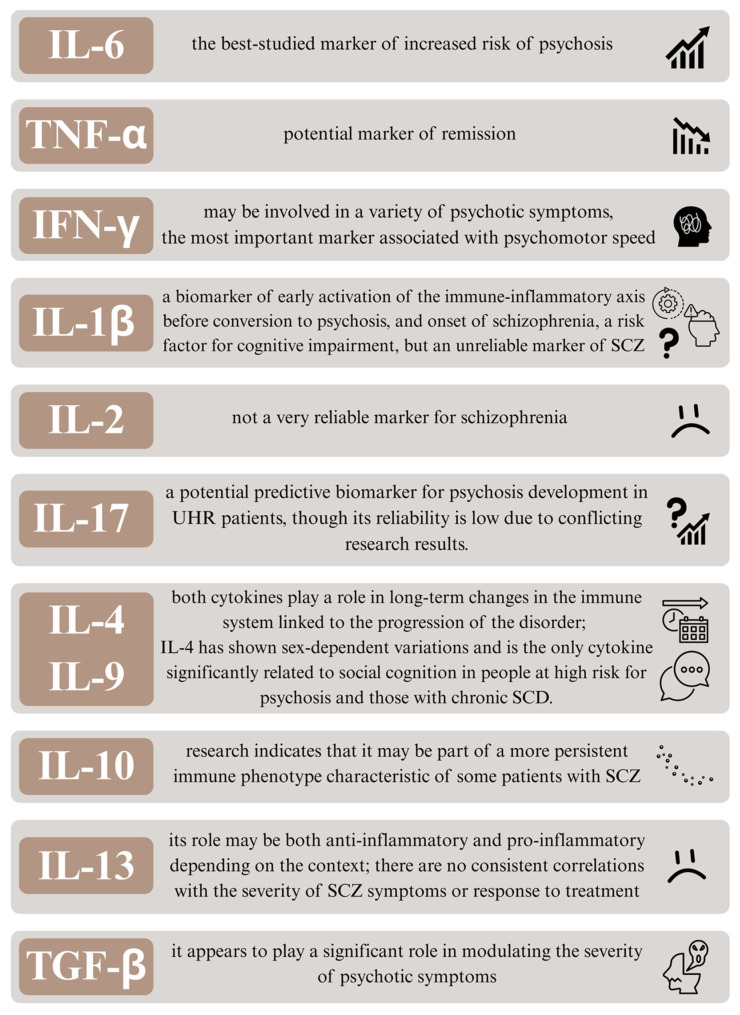
The figure summarizes the most salient information regarding potential inflammatory markers that are useful in the early diagnosis of schizophrenia, monitoring disease progression, and predicting the severity of clinical symptoms. Abbreviations used in the figure: IL-6 (Interleukin-6), TNF-α (Tumor Necrosis Factor alpha), IFN-γ (Interferon gamma), IL-1β (Interleukin-1 beta), IL-2 (Interleukin-2), IL-17 (Interleukin-17), IL-4 (Interleukin-4), IL-9 (Interleukin-9), IL-10 (Interleukin-10), IL-13 (Interleukin-13), IGF-β (Insulin-like Growth Factor beta), SCZ (Schizophrenia), UHR (Ultra-High Risk), SCD (Schizophrenia Clinical Diagnosis).

One of the key anti-inflammatory cytokines attracting increasing interest in the pathogenesis of SCZ is TGF-β. Researchers have noted an increase in TGF-β1 gene expression [165] and protein levels in peripheral blood cells, particularly in FES patients [166]. These changes have been linked to structural brain abnormalities and cognitive deficits, mainly in visual memory and learning [166], as well as with the severity of positive symptoms. In SCZ patients, a strong positive correlation was found between TGF-β levels and the severity of affective symptoms, psychotic symptoms [142], and poorer overall functioning (GAF) [116] (Figure 3).

It was demonstrated that exposure of cortical neurons to IL-6 resulted in decreased expression of genes related to the TGF-β pathway [167]. Nevertheless, it is noteworthy that lower TGF-β levels in later SCZ phases may relate to symptom stabilization, indicating a dynamic role throughout the disease course [144].

Regarding the impact of pharmacotherapy on TGF-β levels, studies have shown that 4–6 months of treatment with olanzapine and risperidone resulted in a decrease in both TGF-β1 [116] and TGF-β2 levels [168]. Interestingly, however, 12 weeks of clozapine treatment (commonly used in TRS) did not result in any significant changes in TGF-β levels [168]. Sun et al. suggest that dysregulation or overactivation of the TGF-β pathway in patients treated with SGAs may contribute to lipid and glucose metabolism disturbances, excessive fat accumulation, and the development of obesity, which in turn plays a critical role in the progression of MetS [169] (Table 3).

## 5. Potential Cross-Talk Between Obesity-Related Inflammation and Schizophrenia

There is growing evidence that obesity and SCZ share a common immunological and inflammatory basis. This may explain their frequent co-occurrence and mutual reinforcement. In conditions of excessive adipose tissue accumulation, there is increased production of IFN-γ, suppression of the Th2 response, and a shift toward a pro-inflammatory Th1 profile [170]. A key mechanism responsible for this shift is elevated leptin levels—a hormone secreted by adipocytes. As adipocyte volume increases, leptin synthesis and secretion also rise. Leptin has immunomodulatory properties: it promotes Th1 responses and inhibits Th2 lymphocyte activity (Figure 2) [15]. Consequently, this leads to increased production of Th1-related pro-inflammatory cytokines such as IFN-γ, IL-2, and TNF-α. At the same time, reduced Th2 activity results in decreased secretion of anti-inflammatory cytokines, including IL-4, interleukin-5 (IL-5), IL-13, and IL-10, fostering a sustained inflammatory state [171]. In the context of obesity and its complications, this immune shift contributes to chronic low-grade inflammation, exacerbation of IR, and lipid metabolism disturbances [172].

Available literature evidence suggests the existence of a so-called vicious cycle between obesity, low-grade inflammation, and SCZ. Importantly, adipose-derived cytokines can influence central immune mechanisms and exacerbate neuroinflammatory processes involved in the pathophysiology of SCZ and potentially contribute to disease severity and progression. Conversely, SCZ treatment (especially long-term antipsychotic pharmacotherapy) is often linked to negative metabolic effects, such as weight gain, dyslipidemia, and IR, thereby exacerbating inflammation [173]. It is increasingly emphasized that the neuroinflammatory background of SCZ and pharmacologically induced metabolic changes may create a vicious cycle mechanism, in which treatment contributes to the worsening of inflammatory and metabolic parameters, which in turn may exacerbate disease symptoms and impair therapeutic response.

Additionally, unhealthy eating habits and gut dysbiosis, frequently observed in patients with SCZ, affect neuroinflammatory pathways and disrupt communication along the gut–brain axis [174]. Emerging evidence further indicates that gut mycobiota alterations, particularly in elderly patients, are linked to systemic immune dysfunction and inflammatory signaling, which may aggravate neuroinflammation and worsen cognitive and psychiatric symptoms. Age-related factors, including immune function, gut motility, and dietary habits, may further increase susceptibility to these microbial disturbances [175]. Although this review does not focus on gut microbiota, the gut–brain axis represents a relevant pathway in the cross-talk between obesity, immune imbalance, and schizophrenia, and warrants further investigation as a potential therapeutic target.

This suggests that each component (obesity, inflammation, SCZ, unhealthy diet, medication) may negatively influence the others, worsening overall health and the course of SCZ. This interplay underscores the necessity of personalized treatment strategies that address psychiatric symptoms and metabolic disturbances. Tailoring antipsychotic therapy to a patient’s inflammatory and metabolic profile may improve clinical outcomes and reduce the risk of comorbidities, providing a more targeted approach to managing SCZ.

The number of studies simultaneously evaluating inflammatory cytokine profiles in patients with SCZ and coexisting obesity remains very limited. Most available publications focus mainly on selected mediators such as IL-6 and TNF-α, leaving other immune components poorly documented. According to van Nimwegen et al., elevated levels of TNF-α and IL-6 in patients with SCZ or schizoaffective disorder who were not taking antipsychotic medication may inactivate phosphatidylinositol-3-kinase—a key enzyme in the insulin receptor pathway—resulting in reduced glucose transport to target tissues via decreased GLUT-4 expression and development of insulin resistance [176]. Other researchers noted that patients with newly diagnosed SCZ, previously untreated with antipsychotics, more frequently exhibited features of MetS and higher IL-6 concentrations [177]. Furthermore, a cross-sectional study by Yuan et al. showed that IL-6 may be used as an effective marker for screening MetS in patients with chronic SCZ [119]. In a study by Chase et al., elevated mRNA levels of pro-inflammatory cytokines IL-6 and TNF-α were observed in patients with SCZ. Importantly, both SCZ diagnosis and BMI ≥ 25 were significant predictors of increased IL-6 and TNF-α mRNA levels [178]. In this context, excess adipose tissue present in some patients constitutes a potential source of IL-6, which may contribute to the chronic inflammatory state observed in SCZ. This study has some limitations due to a significant predominance of men in the SCZ patient group, which may have important implications regarding differences in fat tissue distribution. Interestingly, Soldevila-Matías et al. demonstrated that the combination of baseline IL-6 levels with markers of cardiometabolic activity such as low-density lipoprotein (LDL), systolic blood pressure (SBP), and insulin significantly predicted changes in global cognitive score (GCS) over time in the entire study group, which also included individuals with SCZ and coexisting obesity [179].

Importantly, He et al. showed that elevated IL-6 levels in patients with TRS compared to partially responsive schizophrenia (PRS) patients may be indirectly related to the presence of comorbid obesity, and the inflammatory changes observed in schizophrenic patients may originate from excess adipose tissue, particularly in the male TRS subgroup where a positive correlation between IL-6 levels and BMI was found [180]. In contrast, the study by Mednova et al. found no significant differences in serum levels of pro-inflammatory cytokines TNF-α and IL-6 between SCZ patients with MetS and those without MetS. This indicates that MetS presence was not associated with higher or lower overall levels of these pro-inflammatory cytokines in this particular SCZ patient group [181]. It thus appears that having SCZ itself might affect TNF-α and IL-6 levels, potentially masking additional changes that could result from the presence of MetS. Interesting data comes from Arabska et al., who found no significant differences in IL-6 and TNF-α concentrations between SCZ patients and a healthy control group with comparable metabolic parameters—the average BMI in the SCZ group was 29.0 ± 5.8 kg/m^2^; in the healthy control group, it was 29.5 ± 7.7 kg/m^2^. However, correlation analysis showed that TNF-α was more sensitive to metabolic changes compared to IL-6, and individuals with a higher fat mass index had significantly higher TNF-α levels both in the control group and the entire study sample [182].

It should be emphasized that the number of studies analyzing the mutual relationships between inflammation induced by excess adipose tissue, changes in pro- and anti-inflammatory cytokine levels, and clinical symptoms of SCZ remains limited. This research gap indicates a need for detailed studies considering these variables, which could contribute to identifying common pathophysiological mechanisms and potential therapeutic targets, enabling a more individualized approach to treating obese patients with SCZ and MD.

Another aspect related to obesity in patients with SCZ is their use of antipsychotic medications. It is suspected that weight gain may be caused by drug-induced mechanisms such as 5-hydroxytryptamine 2C (5-HT2C) receptor antagonism; disturbances in the dopaminergic, adrenergic, and histaminergic pathways—particularly involving the H1 receptor; blockade of muscarinic receptors; disruption of the endocannabinoid system, especially the cannabinoid type 1 (CB1) receptors; and altered secretion of insulin and leptin [13].

A conducted meta-analysis showed that nearly all antipsychotic drugs are associated with weight gain, with the exception of amisulpride, aripiprazole, asenapine, sertindole, and ziprasidone. Although direct comparisons between antipsychotics were not tested in this study, the raw data suggest that clozapine and olanzapine are associated with the greatest weight gain after the baseline period. First-generation antipsychotics, such as haloperidol, are also linked to significant weight gain [183].

Another study demonstrated that olanzapine caused the most significant weight gain, followed by asenapine, risperidone, aripiprazole, quetiapine XR, brexpiprazole, cariprazine, and lurasidone. This indicates that even relatively new medications like cariprazine and brexpiprazole resulted in significantly greater weight gain compared to placebo. Only aripiprazole, lurasidone, and quetiapine XR did not lead to clinically significant weight gain (≥7%) [184].

Initially, it was debated whether drug-related weight gain stemmed from increased energy intake or rather reduced energy expenditure. This issue is relevant from both a neurobiological and clinical perspective, as tonic dopamine levels play a key role in initiating motor activity. However, findings from human studies have been inconclusive. For example, it has been shown that olanzapine drives early weight gain through increased food intake, with no evidence of reduced energy expenditure, activity levels, or short-term insulin sensitivity disturbances [185]. In contrast, patients taking clozapine were found to have reduced energy expenditure [186].

It should be emphasized, however, that obesity frequently co-occurs in patients with SCZ, even among those not receiving antipsychotic treatment. A study found that, regardless of medication effects, the longer the duration of SCZ, the higher the BMI and the greater the prevalence of obesity [187].

There is a hypothesis that a specific mechanism in SCZ—namely altered reward anticipation in the striatum related to phasic dopamine responses—may mediate weight gain [188]. Nevertheless, research findings in this area remain inconclusive.

Metformin remains the only drug for which there is some evidence suggesting it may be a well-tolerated and effective agent in preventing weight gain when initiated alongside antipsychotic treatment [189]. However, the most crucial element appears to be a conscious approach to the metabolic risks posed by a given antipsychotic—namely patient education and regular monitoring of body weight and metabolic parameters.

## 6. Anti-Inflammatory Therapies in Schizophrenia

Preclinical studies suggest that anti-inflammatory drugs may potentially not only alleviate psychotic symptoms but also protect against neuronal changes associated with the pathogenesis of SCZ [190]. We have decided to discuss some of these agents.

### 6.1. Aspirin

Aspirin (acetylsalicylic acid) irreversibly inhibits cyclooxygenase-1 (COX-1) activity and modulates cyclooxygenase-2 (COX-2) function, leading to reduced prostaglandin synthesis and, consequently, a decrease in inflammatory responses. It has also been shown that aspirin uncouples oxidative phosphorylation in mitochondria and affects NF-κB signaling pathways—mechanisms that may be relevant to the pathophysiology of SCZ [191]. In two randomized controlled trials (RCTs) using aspirin as an adjunctive therapy in patients with SCZ, improvements were observed in overall outcomes and positive PANSS scores [192,193], although a meta-analysis published in 2019 indicated that the available evidence is limited and inconclusive [194]. Results from another meta-analysis based on two RCTs showed a small, statistically nonsignificant effect of aspirin on total PANSS scores; additionally, the effect was not dependent on CRP levels in patients [195].

### 6.2. N-Acetylcysteine

*N*-acetylcysteine (NAC) has anti-inflammatory properties associated with the reduction in pro-inflammatory cytokines. Other pathways in which NAC may help alleviate SCZ symptoms include regulation of dysregulated neurotransmission (glutamatergic, dopaminergic, gamma-aminobutyric acid (GABA)ergic, serotonergic, cholinergic, and adrenergic pathways) through modulation of synaptic transmission, receptor activity, and transporter function [196]. NAC is also a direct precursor of glutathione, which protects cells from reactive oxygen species [197].

A meta-analysis indicated that NAC improves PANSS scores after 24 weeks of treatment, showing a substantial effect on both negative symptoms and overall PANSS scores [198]. Another study reported that NAC as adjunctive therapy in patients with chronic SCZ significantly improved negative and general symptoms as well as overall clinical improvement (assessed by the CGI scale), with no significant effect on positive symptoms [199]. Additionally, in a 52-week double-blind, placebo-controlled study in early-phase SCZ spectrum patients, NAC significantly improved total PANSS scores, the negative subscale, and thought disorder-related symptoms, but had no effect on the positive PANSS subscale or cognitive performance assessed by BACS. Preliminary analyses suggested that baseline cortical thickness was associated with PANSS improvement in the NAC group, although NAC itself did not induce significant morphological brain changes during the study [200].

### 6.3. Celecoxib

Celecoxib, a widely used selective COX-2 inhibitor, has also been suggested as a potential antipsychotic agent, but the results of existing studies are inconclusive. Several clinical trials have assessed its efficacy as adjunctive therapy. Akhondzadeh et al. reported that in patients with chronic SCZ, an 8-week treatment combining celecoxib (400 mg/day) and risperidone (6 mg/day) was more effective than risperidone alone in improving positive PANSS scores and total PANSS scores [201]. Müller et al. used celecoxib as adjunctive therapy in patients receiving amisulpride and observed significantly better outcomes in both positive and negative symptoms compared to the placebo group, demonstrating for the first time a clear beneficial effect of celecoxib on negative symptoms [202]. However, two other studies did not show a significant therapeutic effect [203].

### 6.4. Minocycline

Minocycline belongs to the tetracycline antibiotic class. In SCZ, its effects are related to anti-inflammatory, neuroprotective properties and inhibition of cytochrome P450 enzymes involved in the metabolism of antipsychotics, including clozapine [204]. Although the mechanisms of minocycline in SCZ are not fully understood, the available literature indicates anti-inflammatory effects and neurotransmitter modulation through the NMDA–NO–cGMP pathway [205]. In participants receiving minocycline as adjunctive therapy, reductions in total, negative, and general symptoms were observed, without significant improvement in positive symptoms or cognitive function [206]. One study suggested that minocycline’s effects may be associated with reduced pro-inflammatory cytokine levels (IL-1β, IL-6, TNF-α) [207]. Liu et al. [208] did not observe changes in IL-1β or TNF-α levels after 16 weeks of treatment but reported interesting correlations between nitric oxide (NO) levels and improvement in negative symptoms (SANS), with lower NO levels correlating with more severe symptoms.

### 6.5. Tocilizumab

Tocilizumab, an anti–IL-6 receptor antibody, is being considered as a potential anti-inflammatory therapy for SCZ, particularly for treatment-resistant symptoms. Clinical studies aim to assess whether modulation of the IL-6 axis can impact positive and negative symptoms as well as cognitive deficits in patients with SCZ, supporting the concept of immunomodulatory strategies in the treatment of this disorder [209].

A small study reported that tocilizumab infusions were well tolerated, with no serious adverse events, and were associated with modest improvements in selected cognitive functions (verbal fluency, digit-symbol coding, composite score) in patients with chronic SCZ. However, no significant therapeutic effects were observed for positive or negative symptoms, nor were changes seen in inflammatory markers (hsCRP, cytokines). These results suggest that tocilizumab’s efficacy may be limited when baseline IL-6 levels are undetectable [210].

A meta-analysis including 70 randomized controlled trials with 4104 participants showed that adjunctive anti-inflammatory therapy outperformed placebo [211]. However, results for anti-inflammatory drugs potentially suitable for broad clinical use were heterogeneous [212]. Differences may be due to patient selection, concomitant antipsychotic treatments, and study duration. Further studies, potentially based on individual metabolic or inflammatory phenotypes, are needed to better tailor treatment for patients with persistent SCZ symptoms.

## 7. Limitations

There are important limitations that should be considered when interpreting the present findings. First and foremost, due to the review-based nature of this article, no systematic inclusion or exclusion criteria were applied, nor was a critical assessment of methodological quality conducted. The included studies varied in sample size and methodological rigor, which impacts the consistency of conclusions.

Discrepancies in the reported results may arise from differences in the sensitivity and specificity of analytical methods, clinical heterogeneity of patients (e.g., disease stage, deficit subtype, first hospitalization), the immunomodulatory effects of antipsychotic medications (e.g., olanzapine and risperidone may reduce IL-6 levels), metabolic factors (obesity, metabolic syndrome), lifestyle variables (smoking, diet, physical activity), and insufficient control of confounding factors such as age, sex, or duration of illness.

Despite these limitations, many studies indicate a relationship between the severity of inflammation and both the intensity of clinical symptoms and treatment effectiveness in patients with SCZ. Further research employing standardized measurement methods, well-characterized study populations, and long-term follow-up is required.

## 8. Summary

Excess body weight and the associated chronic inflammation are playing an increasingly important role in the pathophysiology of SCZ. Adipose tissue, functioning as an endocrine organ, influences the immune system by amplifying inflammatory responses, which may contribute to the neuroimmunological disturbances observed in individuals with SCZ. Cytokine profiling, encompassing both pro-inflammatory markers (e.g., IL-6, TNF-α, CRP) and anti-inflammatory markers, may allow for the identification of specific immunological patterns that differentiate disease course, severity of psychotic symptoms, cognitive deficits, and structural brain alterations.

Assessing the balance between Th1 and Th2 immune responses in patients with excessive adipose tissue may provide new insights into neuro-inflammatory mechanisms and their relevance to SCZ. Importantly, measuring inflammatory markers (e.g., in blood serum) and evaluating fat mass (via bioelectrical impedance analysis or dual-energy X-ray absorptiometry) are relatively simple, minimally invasive methods readily available in routine clinical practice. This enables early identification of high-risk patients and the personalization of treatment based on metabolic and immunological profiles, potentially improving prognosis and reducing the risk of relapse and hospitalization.

It is also worth emphasizing that weight gain and its metabolic consequences are often insufficiently monitored during antipsychotic therapy, despite their significant impact on patients’ somatic and mental health. Incorporating the assessment of adipose tissue-related inflammation into standard diagnostics and care for patients with SCZ could be an important step toward a more individualized and interdisciplinary therapeutic approach.

## Figures and Tables

**Table 1:** Cytokine-specific literature search results (PubMed).

Cytokine	Search String Used	Search Period	Number of Results	Original Studies Included
IL-6	“IL-6 schizophrenia”	2018–2025	440	35
TNF-α	“TNF schizophrenia”	2023–2025	106	21
IL-1β	“IL-1β schizophrenia”	2022–2025	127	15
IL-2	“IL-2 schizophrenia”	2018–2025	87	17
IFN-γ	“IFN-γ schizophrenia”	2018–2025	98	19
IL-17	“IL-17 schizophrenia”	2018–2025	66	19
IL-4	“IL-4 schizophrenia”	2018–2025	98	23
IL-10	“IL-10 schizophrenia”	2018–2025	159	34
IL-13	“IL-13 schizophrenia”	2018–2025	18	6
TGF-β	“TGF-β schizophrenia”	2018–2025	45	7

Abbreviations and symbols used in the table: Interleukin-6 (IL-6), Tumor Necrosis Factor-alpha (TNF-α), Interleukin-1 beta (IL-1β), Interleukin-2 (IL-2), Interferon-gamma (IFN-γ), Interleukin-17 (IL-17), Interleukin-4 (IL-4), Interleukin-10 (IL-10), Interleukin-13 (IL-13), Transforming Growth Factor-beta (TGF-β).

**Table 2:** The table provides an overview of the associations between elevated concentrations of specific pro- and anti-inflammatory cytokines and clinical domains such as positive symptoms, negative symptoms, and cognitive performance in individuals diagnosed with schizophrenia (SCZ).

Cytokine		Positive Symptoms	Negative Symptoms	Cognitive Abilities
Pro-Inflammatory	**IL-6**	↑ [128]	↑ (chronic SCZ) [37] ↓ (EOS) [129]	↓ [34,128,130] 0 [37]
	**TNF-α**	↑ [54] 0 [60]	↓ [66] 0 [60]	contradictory results: ↑/0/↓ [57,61,63,64]
	**IL-1β**	↑ [85] 0 [43]	0 [43]	↓ [86] 0 [43]
	**IL-2**	↑ [60,98]	0 [23]	↓ real-world functioning [131]
	**IFN-γ**	↓ [109] 0 [115]	↑ [108,116] 0 [115]	↑ [108,109]
	**IL-17**	↑ [123] 0 [132]	0 [122,132]	↑ [125]
Anti-Inflammatory	**IL-4**	↓ [133,134] 0 [60]	↑ [135] 0 [60,134]	↑ [133,136,137]
	**IL-10**	↑ [60] 0 [123,133,138]	contradictory results: ↑ [60,115,139,140] 0 [123,133,138] ↓ [141]	0 [133,138]
	**IL-13**	0 [115]	0 [115]	↑ [133]
	**TGF-β**	↑ [142]	no studies available	no studies available

Symbols used in the table: ↑ (greater symptom severity or better cognitive abilities), ↓ (less symptom severity or poorer cognitive abilities), 0 (no correlation with symptom severity or cognitive abilities). Abbreviations used in the table: IL-6 (Interleukin-6), TNF-α (Tumor Necrosis Factor alpha), IL-1β (Interleukin-1 beta), IL-2 (Interleukin-2), IFN-γ (Interferon gamma), IL-17 (Interleukin-17), IL-4 (Interleukin-4), IL-10 (Interleukin-10), IL-13 (Interleukin-13), TGF-β (Transforming Growth Factor Beta), EOS (Early-Onset Schizophrenia).

**Table 3:** Effects Of Antipsychotic Pharmacotherapy On Cytokine Levels In Schizophrenia.

Group	Cytokine	Short-Term Treatment (Up To 10 Weeks)	Medium- To Long-Term Treatment (≥6 Months)	Exemplary Drugs And Clinical Or Metabolic Notes
Pro-Inflammatory	IL-6	Levels ↓ with escitalopram, associated with improvement in negative and cognitive PANSS symptoms [48]. Levels ↑ with olanzapine, correlated with IR and adipose inflammation. Haloperidol did not ↑ IL-6 in adipose tissue [49].	Levels ↓ with risperidone, olanzapine, aripiprazole, and quetiapine, associated with improved working memory and verbal learning [47].	Short-term reduction may support cognitive improvement, whereas drug-specific increases (e.g., olanzapine) are linked to metabolic risk.
	TNF-α	Levels ↑ after paliperidone (alone or with sertraline) and after olanzapine [60,65]. Aripiprazole decreased TNF-α [67].	–	Aripiprazole also ↓ leptin and improved body weight, BMI, and body fat percentage.
	IL-1β	Levels remained elevated after four weeks of atypical antipsychotic therapy [53].	Levels remained Ns after twelve months of aripiprazole or olanzapine, regardless of sex [88].	IL-1β levels appear resistant to both short- and long-term pharmacological intervention.
	IL-2	Levels ↓ with risperidone, olanzapine, and haloperidol. Clozapine showed Ns change [90].	Levels remained Ns after fifty-two weeks of aripiprazole or olanzapine [88]. Levels ↑ after five years despite clinical improvement [92].	Findings suggest a biphasic effect: early ↓, medium-term stabilization, and long-term rebound.
	IFN-γ	Levels ↓ after treatment with risperidone, olanzapine, or aripiprazole [97], associated with improvement of negative symptoms and functioning [116].	Levels remained Ns after twelve months of amisulpride, aripiprazole, or olanzapine [88].	Short-term ↓ may reflect acute-phase response, whereas long-term findings are inconsistent.
	IL-17	Levels showed a trend toward ↓ after ten weeks of risperidone monotherapy, but changes were Ns [97,118].	Levels ↓ after six months of therapy [116].	IL-17 may serve as a marker of the acute phase, normalizing with longer pharmacotherapy.
Anti-Inflammatory	IL-4	Levels remained Ns after four weeks of risperidone treatment [92].	Levels ↓ after one year in patients with predominant depressive symptoms [148]. Sex-dependent differences were observed after fifty-two weeks (increase in women, decrease in men) [88]. Levels remained Ns after five years of risperidone treatment [92].	Findings highlight the importance of considering sex differences and depressive symptom profile in interpreting IL-4 responses.
	IL-10	Levels ↑ with olanzapine and clozapine. Levels further increased after six weeks of olanzapine [60,138,161].	–	Antipsychotic therapy, especially olanzapine and clozapine, is associated with up-regulation of IL-10.
	IL-13	–	–	No significant association with treatment response [115].
	TGF-β	Levels remained Ns after twelve weeks of clozapine [168].	Levels ↓ after four to six months of olanzapine and risperidone [116,168].	Dysregulation of the TGF-β pathway under SGAs may contribute to obesity and MetS.

Abbreviations used in the table: IL-6 (Interleukin-6), TNF-α (Tumor Necrosis Factor alpha), IL-1β (Interleukin-1 beta), IL-2 (Interleukin-2), IFN-γ (Interferon gamma), IL-17 (Interleukin-17), IL-4 (Interleukin-4), IL-10 (Interleukin-10), IL-13 (Interleukin-13), TGF-β (Transforming Growth Factor beta), PANSS (Positive and Negative Syndrome Scale), BMI (Body Mass Index), SGAs (Second-Generation Antipsychotics), IR (insulin resistance), MetS (metabolic syndrome); Arrows indicate the direction of change in cytokine levels after treatment compared to baseline: ↓ decrease, ↑ increase; Ns not significant; “–” = not reported in the reviewed studies.

## References

[1] Nawaz R., Gul S., Amin R., Huma T., Al Mughairbi F. (2020). Overview of Schizophrenia Research and Treatment in Pakistan. Heliyon.

[2] Na K.-S., Jung H.-Y., Kim Y.-K. (2014). The Role of Pro-Inflammatory Cytokines in the Neuroinflammation and Neurogenesis of Schizophrenia. Prog. Neuropsychopharmacol. Biol. Psychiatry.

[3] Manu P., Dima L., Shulman M., Vancampfort D., De Hert M., Correll C.U. (2015). Weight Gain and Obesity in Schizophrenia: Epidemiology, Pathobiology, and Management. Acta Psychiatr. Scand..

[4] Galic S., Oakhill J.S., Steinberg G.R. (2010). Adipose Tissue as an Endocrine Organ. Mol. Cell. Endocrinol..

[5] Saddichha S., Manjunatha N., Ameen S., Akhtar S. (2007). Effect of Olanzapine, Risperidone, and Haloperidol Treatment on Weight and Body Mass Index in First-Episode Schizophrenia Patients in India: A Randomized, Double-Blind, Controlled, Prospective Study. J. Clin. Psychiatry.

[6] Strassnig M., Caceda R., Newcomer J., Harvey P. (2012). Cognitive Deficits, Obesity and Disability in Schizophrenia. Transl. Neurosci..

[7] Chen Y.Q., Li X.R., Zhang L., Zhu W.B., Wu Y.Q., Guan X.N., Xiu M.H., Zhang X.Y. (2021). Therapeutic Response Is Associated With Antipsychotic-Induced Weight Gain in Drug-Naive First-Episode Patients With Schizophrenia: An 8-Week Prospective Study. J. Clin. Psychiatry.

[8] Doane M.J., Bessonova L., Friedler H.S., Mortimer K.M., Cheng H., Brecht T., O’Sullivan A.K., Cummings H., McDonnell D., Meyer J.M. (2022). Weight Gain and Comorbidities Associated with Oral Second-Generation Antipsychotics: Analysis of Real-World Data for Patients with Schizophrenia or Bipolar I Disorder. BMC Psychiatry.

[9] McIntyre R.S., McCann S.M., Kennedy S.H. (2001). Antipsychotic Metabolic Effects: Weight Gain, Diabetes Mellitus, and Lipid Abnormalities. Can. J. Psychiatry.

[10] Leucht S., Corves C., Arbter D., Engel R.R., Li C., Davis J.M. (2009). Second-Generation versus First-Generation Antipsychotic Drugs for Schizophrenia: A Meta-Analysis. Lancet.

[11] Allison D.B., Mentore J.L., Heo M., Chandler L.P., Cappelleri J.C., Infante M.C., Weiden P.J. (1999). Antipsychotic-Induced Weight Gain: A Comprehensive Research Synthesis. Am. J. Psychiatry.

[12] McWhinney S.R., Brosch K., Calhoun V.D., Crespo-Facorro B., Crossley N.A., Dannlowski U., Dickie E., Dietze L.M.F., Donohoe G., Du Plessis S. (2022). Obesity and Brain Structure in Schizophrenia—ENIGMA Study in 3021 Individuals. Mol. Psychiatry.

[13] Panariello F., De Luca V., De Bartolomeis A. (2011). Weight Gain, Schizophrenia and Antipsychotics: New Findings from Animal Model and Pharmacogenomic Studies. Schizophr. Res. Treat..

[14] Khanna D., Khanna S., Khanna P., Kahar P., Patel B.M. (2022). Obesity: A Chronic Low-Grade Inflammation and Its Markers. Cureus.

[15] Brito Díaz B., Marcelino Rodríguez I., Almeida González D., Rodríguez Pérez M.D.C., Cabrera De León A. (2014). An Overview of Leptin and the Th1/Th2 Balance. Open J. Immunol..

[16] Tsai S., Sajatovic M., Hsu J., Chung K., Chen P., Huang Y. (2020). Body Mass Index, Residual Psychotic Symptoms, and Inflammation Associated with Brain Volume Reduction in Older Patients with Schizophrenia. Int. J. Geriatr. Psychiatry.

[17] Yeşilkaya Ü.H., Şen M., BalcIoğlu Y.H., Gokçay H., Çelikkıran P., Kırlıoğlu Balcıoğlu S., Karamustafalıoğlu N. (2024). Evaluation of the Correlation Between Peripheral Inflammatory Markers and Suicide Risk in Drug-Naive First-Episode Schizophrenia. Arch. Neuropsychiatry.

[18] Baldini V., Gnazzo M., Varallo G., Atti A.R., De Ronchi D., Fiorillo A., Plazzi G. (2025). Inflammatory Markers and Suicidal Behavior: A Comprehensive Review of Emerging Evidence. Ann. Gen. Psychiatry.

[19] Sun Y., Wu D., Yang X., Tang B., Xia C., Luo C., Gong Q., Lui S., Hu N. (2024). The Associations of Peripheral Interleukin Alterations and Hippocampal Subfield Volume Deficits in Schizophrenia. Cereb. Cortex.

[20] Li M., Liu Y., Sun M., Yang Y., Zhang L., Liu Y., Li F., Liu H. (2024). SEP-363856 Exerts Neuroprotection through the PI3K/AKT/GSK-3β Signaling Pathway in a Dual-hit Neurodevelopmental Model of Schizophrenia-like Mice. Drug Dev. Res..

[21] Michalczyk A., Tyburski E., Podwalski P., Waszczuk K., Rudkowski K., Kucharska-Mazur J., Mak M., Rek-Owodziń K., Plichta P., Bielecki M. (2024). Greater Methylation of the IL-6 Promoter Region Is Associated with Decreased Integrity of the Corpus Callosum in Schizophrenia. J. Psychiatr. Res..

[22] Liu X., Ling Z., Cheng Y., Wu L., Shao L., Gao J., Lei W., Zhu Z., Ding W., Song Q. (2024). Oral Fungal Dysbiosis and Systemic Immune Dysfunction in Chinese Patients with Schizophrenia. Transl. Psychiatry.

[23] Lesh T.A., Careaga M., Rose D.R., McAllister A.K., Van De Water J., Carter C.S., Ashwood P. (2018). Cytokine Alterations in First-Episode Schizophrenia and Bipolar Disorder: Relationships to Brain Structure and Symptoms. J. Neuroinflamm..

[24] Zhang Y., Wang J., Ye Y., Zou Y., Chen W., Wang Z., Zou Z. (2023). Peripheral Cytokine Levels across Psychiatric Disorders: A Systematic Review and Network Meta-Analysis. Prog. Neuropsychopharmacol. Biol. Psychiatry.

[25] Guo J., Liu C., Wang Y., Feng B., Zhang X. (2015). Role of T Helper Lymphokines in the Immune-Inflammatory Pathophysiology of Schizophrenia: Systematic Review and Meta-Analysis. Nord. J. Psychiatry.

[26] Borovcanin M., Jovanovic I., Radosavljevic G., Djukic Dejanovic S., Bankovic D., Arsenijevic N., Lukic M.L. (2012). Elevated Serum Level of Type-2 Cytokine and Low IL-17 in First Episode Psychosis and Schizophrenia in Relapse. J. Psychiatr. Res..

[27] Noto C., Ota V.K., Santoro M.L., Ortiz B.B., Rizzo L.B., Higuchi C.H., Cordeiro Q., Belangero S.I., Bressan R.A., Gadelha A. (2015). Effects of Depression on the Cytokine Profile in Drug Naïve First-Episode Psychosis. Schizophr. Res..

[28] Reale M., Costantini E., Greig N.H. (2021). Cytokine Imbalance in Schizophrenia. From Research to Clinic: Potential Implications for Treatment. Front. Psychiatry.

[29] Gkrinia E.M.M., Belančić A. (2025). The Mechanisms of Chronic Inflammation in Obesity and Potential Therapeutic Strategies: A Narrative Review. Curr. Issues Mol. Biol..

[30] Wang Y., Fan L., He Y., Yuan L., Li Z., Zheng W., Tang J., Li C., Jin K., Liu W. (2024). Compensatory Thickening of Cortical Thickness in Early Stage of Schizophrenia. Cereb. Cortex.

[31] Zhang Y., Catts V.S., Sheedy D., McCrossin T., Kril J.J., Shannon Weickert C. (2016). Cortical Grey Matter Volume Reduction in People with Schizophrenia Is Associated with Neuro-Inflammation. Transl. Psychiatry.

[32] Fond G., Lançon C., Korchia T., Auquier P., Boyer L. (2020). The Role of Inflammation in the Treatment of Schizophrenia. Front. Psychiatry.

[33] Corley E., Gleeson C., Godfrey E., Cowman M., Patlola S.R., Cannon D.M., McKernan D.P., Kelly J.P., Hallahan B., McDonald C. (2024). Corpus Callosum Microstructural Organization Mediates the Effects of Physical Neglect on Social Cognition in Schizophrenia. Prog. Neuropsychopharmacol. Biol. Psychiatry.

[34] Cui L.-J., Cai L.-L., Na W.-Q., Jia R.-L., Zhu J.-L., Pan X. (2024). Interaction between Serum Inflammatory Cytokines and Brain-Derived Neurotrophic Factor in Cognitive Function among First-Episode Schizophrenia Patients. World J. Psychiatry.

[35] Zhang Y., Yin J., Yan H., Yan L., Li Y., Zhang C., Li Y., Liu B., Lin J., Zhang L. (2023). Correlations between Omega-3 Fatty Acids and Inflammatory/Glial Abnormalities: The Involvement of the Membrane and Neurotransmitter Dysfunction in Schizophrenia. Front. Cell. Neurosci..

[36] Taha M.A., AL-maqati T.N., Alnaam Y.A., Alharbi S.S., Khaneen R., Almutairi H., AL-harbi M. (2022). The Association between Brain-Derived Neurotrophic Factor (BDNF) Protein Level and Body Mass Index. Medicina.

[37] Corsi-Zuelli F., Donohoe G., Griffiths S.L., Del-Ben C.M., Watson A.J., Burke T., Lalousis P.A., McKernan D., Morris D., Kelly J. (2025). Depressive and Negative Symptoms in the Early and Established Stages of Schizophrenia: Integrating Structural Brain Alterations, Cognitive Performance, and Plasma Interleukin 6 Levels. Biol. Psychiatry Glob. Open Sci..

[38] Herniman S.E., Wood S.J., Khandaker G., Dazzan P., Pariante C.M., Barnes N.M., Krynicki C.R., Nikkheslat N., Vincent R.C., Roberts A. (2023). Network Analysis of Inflammation and Symptoms in Recent Onset Schizophrenia and the Influence of Minocycline during a Clinical Trial. Transl. Psychiatry.

[39] Goldsmith D.R., Haroon E., Miller A.H., Strauss G.P., Buckley P.F., Miller B.J. (2018). TNF-α and IL-6 Are Associated with the Deficit Syndrome and Negative Symptoms in Patients with Chronic Schizophrenia. Schizophr. Res..

[40] Miyano T., Hirouchi M., Yoshimura N., Hattori K., Mikkaichi T., Kiyosawa N. (2024). Plasma microRNAs Associate Positive, Negative, and Cognitive Symptoms with Inflammation in Schizophrenia. Int. J. Mol. Sci..

[41] Lv D., Xiao B., Liu H., Wang L., Li Y., Zhang Y.H., Jin Q. (2024). Enhanced NMDA Receptor Pathway and Glutamate Transmission in the Hippocampal Dentate Gyrus Mediate the Spatial Learning and Memory Impairment of Obese Rats. Pflüg. Arch. Eur. J. Physiol..

[42] Sales A.J., Gobira P.H., Pedrazzi J.F.C., Silveira J.R., Del Bel E., Gomes F.V., Guimarães F.S. (2024). Doxycycline Diminishes the Rewarding and Psychomotor Effects Induced by Morphine and Cocaine. Prog. Neuropsychopharmacol. Biol. Psychiatry.

[43] Cheng X., Xie Y., Wang A., Zhu C., Yan F., Pei W., Zhang X. (2023). Correlation between Elevated Serum Interleukin-1β, Interleukin-16 Levels and Psychiatric Symptoms in Patients with Schizophrenia at Different Stages. BMC Psychiatry.

[44] Rangel S.C., Da Silva M.D., Natrielli Filho D.G., Santos S.N., Do Amaral J.B., Victor J.R., Silva K.C.N., Tuleta I.D., França C.N., Shio M.T. (2024). HERV-W Upregulation Expression in Bipolar Disorder and Schizophrenia: Unraveling Potential Links to Systemic Immune/Inflammation Status. Retrovirology.

[45] Enokida T., Hattori K., Okabe K., Noda T., Ota M., Sato N., Ogawa S., Tatsumi M., Hoshino M., Kunugi H. (2024). Possible Association of Elevated CSF IL -6 Levels with Anxiety and Frustration in Psychiatric Disorders. Psychiatry Clin. Neurosci..

[46] Sahoo S., Kale A., Basu D., Minz R.W. (2023). Is There Any Association between Cognitive Deficits and Immune Markers in Acute and Transient Psychotic Disorders? A Pilot Study. Asian J. Psychiatry.

[47] Zhuo C., Hu S., Chen G., Yang L., Cai Z., Tian H., Jiang D., Chen C., Wang L., Ma X. (2023). Low-Dose Lithium Adjunct to Atypical Antipsychotic Treatment Nearly Improved Cognitive Impairment, Deteriorated the Gray-Matter Volume, and Decreased the Interleukin-6 Level in Drug-Naive Patients with First Schizophrenia Symptoms: A Follow-up Pilot Study. Schizophrenia.

[48] Ding N., Li Z., Liu Z. (2018). Escitalopram Augmentation Improves Negative Symptoms of Treatment Resistant Schizophrenia Patients—A Randomized Controlled Trial. Neurosci. Lett..

[49] Calevro A., Cotel M.-C., Natesan S., Modo M., Vernon A.C., Mondelli V. (2018). Effects of Chronic Antipsychotic Drug Exposure on the Expression of Translocator Protein and Inflammatory Markers in Rat Adipose Tissue. Psychoneuroendocrinology.

[50] Li H., Peng S., Li S., Liu S., Lv Y., Yang N., Yu L., Deng Y.-H., Zhang Z., Fang M. (2019). Chronic Olanzapine Administration Causes Metabolic Syndrome through Inflammatory Cytokines in Rodent Models of Insulin Resistance. Sci. Rep..

[51] Shen B., Lu R., Lv M., Chen J., Li J., Long J., Cai H., Su L., Gong Z. (2024). Association between the Levels of Toxic Heavy Metals and Schizophrenia in the Population of Guangxi, China: A Case-Control Study. Environ. Pollut..

[52] Ray A., Birdi A., Nebhinani N., Banerjee M., Sharma P., Sharma S., Suthar N., Janu V.C., Yadav D. (2025). Correlation Between Severity of Schizophrenia with Certain Trace Elements and TNF-α Gene Expression and Its Circulatory Level in the Population of Western India. Biol. Trace Elem. Res..

[53] Hatzimanolis A., Foteli S., Xenaki L.-A., Selakovic M., Dimitrakopoulos S., Vlachos I., Kosteletos I., Soldatos R.-F., Gazouli M., Chatzipanagiotou S. (2024). Elevated Serum Kynurenic Acid in Individuals with First-Episode Psychosis and Insufficient Response to Antipsychotics. Schizophrenia.

[54] Wang X., Chen W., Gou M., Li W., Li N., Tong J., Zhou Y., Xie T., Yu T., Feng W. (2024). Relationship between Plasma TNF-α Levels and Agitation Symptoms in First Episode Patients with Schizophrenia. BMC Psychiatry.

[55] Serazetdinova V.S., Petrova N.N., Dorofeykov V.V., Mayorova M.A. (2025). Clinical and Immunological Relationships in Patients with Early Schizophrenia. SS Korsakov J. Neurol. Psychiatry.

[56] Çiftci H., Aşut G., Kaya H., Çakmak I.B., Aydıner Yılmaz M., Çöpür A., Çalcı E., Fırat Oğuz E., Turhan T., Göka E. (2024). Neutrophil Gelatinase-Associated Lipocalin (NGAL) and Inflammatory Markers in Schizophrenia: A Comparative Analysis of Drug-Naive Schizophrenia Patients, Remitted Patients, and Healthy Controls. J. Psychiatr. Res..

[57] Asada R., Hori H., Gotoh L., Yasumatsu K., Iida H., Kawasaki H. (2024). Lower Plasma Tumor Necrosis Factor-α Is Associated with Symptomatic Remission in Patients with Schizophrenia. J. Psychiatr. Res..

[58] Su L., Liu X., Li Y., Yuan H., Li Q., Li C. (2023). Comparison of Olfactory Function, Cognitive Function and Serum Tumor Necrosis Factor-α between Bipolar and Schizophrenic Patients in the Remission Stage. BMC Psychiatry.

[59] Zheng Y., Zhang Q., Zhou X., Yao L., Zhu Q., Fu Z. (2023). Altered Levels of Cytokine, T- and B-Lymphocytes, and PD-1 Expression Rates in Drug-Naïve Schizophrenia Patients with Acute Phase. Sci. Rep..

[60] Zhao X., Zhu W., Bu Y., Li J., Hao Y., Bi Y. (2024). Effects of 6-Week Olanzapine Treatment on Serum IL-2, IL-4, IL-8, IL-10, and TNF-α Levels in Drug-Naive Individuals with First-Episode Schizophrenia. BMC Psychiatry.

[61] Pavlovic M., Babic D., Rastovic P., Arapovic J., Martinac M., Jakovac S., Barbaric R. (2023). Association of Tumor Necrosis Factor-Alpha with Psychopathology in Patients with Schizophrenia. Acta Medica Okayama.

[62] Ermakov E., Melamud M., Boiko A., Kamaeva D., Ivanova S., Nevinsky G., Buneva V. (2023). Association of Peripheral Inflammatory Biomarkers and Growth Factors Levels with Sex, Therapy and Other Clinical Factors in Schizophrenia and Patient Stratification Based on These Data. Brain Sci..

[63] Sapienza J., Agostoni G., Comai S., Nasini S., Dall’Acqua S., Sut S., Spangaro M., Martini F., Bechi M., Buonocore M. (2024). Neuroinflammation and Kynurenines in Schizophrenia: Impact on Cognition Depending on Cognitive Functioning and Modulatory Properties in Relation to Cognitive Remediation and Aerobic Exercise. Schizophr. Res. Cogn..

[64] Wang T.-Y., Chang Y.-H., Lee S.-Y., Chang H.H., Tsai T.-Y., Tseng H.-H., Wang S.-M., Chen P.S., Chen K.C., Lee I.H. (2025). Transdiagnostic Features of Inflammatory Markers and Executive Function across Psychiatric Disorders. J. Psychiatr. Res..

[65] Gu M., Pi Z., Zhu L., Zhang J. (2025). Effect of Paliperidone Combined with Sertraline in the Treatment of Schizophrenia and Its Influence on Serum Neurofunctional Related Factors. Alpha Psychiatry.

[66] Yang H., Peng R., Yang M., Zhang J., Shi Z., Zhang X. (2024). Association between Elevated Serum Matrix Metalloproteinase-2 and Tumor Necrosis Factor-α, and Clinical Symptoms in Male Patients with Treatment-Resistant and Chronic Medicated Schizophrenia. BMC Psychiatry.

[67] Sobiś J., Kunert Ł., Rykaczewska-Czerwińska M., Świętochowska E., Gorczyca P. (2022). The Effect of Aripiprazole on Leptin Levels of Patients with Chronic Schizophrenia and a Comparison of Leptin, Acute Phase Protein, and Cytokine Levels with Regard to Body Mass and Body Composition Indexes. Endokrynol. Pol..

[68] Fond G., Lançon C., Auquier P., Boyer L. (2018). C-Reactive Protein as a Peripheral Biomarker in Schizophrenia. An Updated Systematic Review. Front. Psychiatry.

[69] Hsuchou H., Kastin A.J., Mishra P.K., Pan W. (2012). C-Reactive Protein Increases BBB Permeability: Implications for Obesity and Neuroinflammation. Cell Physiol. Biochem..

[70] Joseph J., Depp C., Martin A.S., Daly R.E., Glorioso D.K., Palmer B.W., Jeste D.V. (2015). Associations of High Sensitivity C-Reactive Protein Levels in Schizophrenia and Comparison Groups. Schizophr. Res..

[71] Johnsen E., Fathian F., Kroken R.A., Steen V.M., Jørgensen H.A., Gjestad R., Løberg E.-M. (2016). The Serum Level of C-Reactive Protein (CRP) Is Associated with Cognitive Performance in Acute Phase Psychosis. BMC Psychiatry.

[72] Dickerson F., Stallings C., Origoni A., Schroeder J., Katsafanas E., Schweinfurth L., Savage C., Khushalani S., Yolken R. (2015). Inflammatory Markers in Recent Onset Psychosis and Chronic Schizophrenia. Schizophr. Bull..

[73] Fan X., Goff D.C., Henderson D.C. (2007). Inflammation and Schizophrenia. Expert Rev. Neurother..

[74] Dickerson F., Stallings C., Origoni A., Boronow J., Yolken R. (2007). C-Reactive Protein Is Associated with the Severity of Cognitive Impairment but Not of Psychiatric Symptoms in Individuals with Schizophrenia. Schizophr. Res..

[75] Boozalis T., Teixeira A.L., Cho R.Y.-J., Okusaga O. (2018). C-Reactive Protein Correlates with Negative Symptoms in Patients with Schizophrenia. Front. Public Health.

[76] Barzilay R., Lobel T., Krivoy A., Shlosberg D., Weizman A., Katz N. (2016). Elevated C-Reactive Protein Levels in Schizophrenia Inpatients Is Associated with Aggressive Behavior. Eur. Psychiatry.

[77] Misiak B., Stańczykiewicz B., Kotowicz K., Rybakowski J.K., Samochowiec J., Frydecka D. (2018). Cytokines and C-Reactive Protein Alterations with Respect to Cognitive Impairment in Schizophrenia and Bipolar Disorder: A Systematic Review. Schizophr. Res..

[78] Dickerson F., Yolken R. (2017). 12. C-Reactive Protein and Suicide Attempts in Schizophrenia. Schizophr. Bull..

[79] Gonzalez-Blanco L., Garcia-Portilla M.P., Garcia-Alvarez L., de la Fuente-Tomas L., Garcia C.I., Saiz P.A., Bobes J. (2018). Elevated C-Reactive Protein as a Predictor of a Random One-Year Clinical Course in the First Ten Years of Schizophrenia. Psychiatry Res..

[80] Metcalf S.A., Jones P.B., Nordstrom T., Timonen M., Mäki P., Miettunen J., Jääskeläinen E., Järvelin M.-R., Stochl J., Murray G.K. (2017). Serum C-Reactive Protein in Adolescence and Risk of Schizophrenia in Adulthood: A Prospective Birth Cohort Study. Brain Behav. Immun..

[81] Koskuvi M., Pörsti E., Hewitt T., Räsänen N., Wu Y.-C., Trontti K., McQuade A., Kalyanaraman S., Ojansuu I., Vaurio O. (2024). Genetic Contribution to Microglial Activation in Schizophrenia. Mol. Psychiatry.

[82] Jia C., Zhang M., Wu X., Zhang X., Lv Z., Zhao K., Zhang J., Su Y., Zhu F. (2025). HERV-W Env Induces Neuron Pyroptosis via the NLRP3–CASP1–GSDMD Pathway in Recent-Onset Schizophrenia. Int. J. Mol. Sci..

[83] Li H., Chen W., Gou M., Li W., Tong J., Zhou Y., Xie T., Yu T., Feng W., Li Y. (2022). The Relationship between TLR4/NF-κB/IL-1β Signaling, Cognitive Impairment, and White-Matter Integrity in Patients with Stable Chronic Schizophrenia. Front. Psychiatry.

[84] Zhao X., Liu Y., Long Q., Zhang Y., You X., Guo Z., Cao X., Yu L., Qin F., Teng Z. (2023). Abnormal Expression of miR-3653-3p, Caspase 1, IL-1β in Peripheral Blood of Schizophrenia. BMC Psychiatry.

[85] Yan F., Meng X., Cheng X., Pei W., Chen Y., Chen L., Zheng M., Shi L., Zhu C., Zhang X. (2023). Potential Role between Inflammatory Cytokines and Tie-2 Receptor Levels and Clinical Symptoms in Patients with First-Episode Schizophrenia. BMC Psychiatry.

[86] Baek S.-H., Kim H., Kim J.-W., Ryu S., Lee J.-Y., Kim J.-M., Shin I.-S., Kim S.-W. (2022). Association between Peripheral Inflammatory Cytokines and Cognitive Function in Patients with First-Episode Schizophrenia. J. Pers. Med..

[87] Tian Y., Li Z., Zhang Y., Tang P., Zhuang Y., Liu L., Fan H., Yao X., Li W., Xia L. (2025). Sex Differences in the Association between Metabolic Disorder and Inflammatory Cytokines in Han Chinese Patients with Chronic Schizophrenia. Front. Psychiatry.

[88] Ratke I., Torsvik A., Bartz-Johannessen C.A., Fathian F., Joa I., Reitan S.M.K., Løberg E.M., Rettenbacher M., Skrede S., Steen V.M. (2025). Sex Differences in the Peripheral Levels of Cytokines during 12-Month Antipsychotic Treatment in a Drug-Naïve Schizophrenia Spectrum Cohort. Brain Behav. Immun. Health.

[89] Çakici N., Sutterland A.L., Penninx B.W.J.H., Dalm V.A., De Haan L., Van Beveren N.J.M. (2020). Altered Peripheral Blood Compounds in Drug-Naïve First-Episode Patients with Either Schizophrenia or Major Depressive Disorder: A Meta-Analysis. Brain Behav. Immun..

[90] Romeo B., Brunet-Lecomte M., Martelli C., Benyamina A. (2018). Kinetics of Cytokine Levels during Antipsychotic Treatment in Schizophrenia: A Meta-Analysis. Int. J. Neuropsychopharmacol..

[91] León-Ortiz P., Rivera-Chávez L.F., Torres-Ruíz J., Reyes-Madrigal F., Carrillo-Vázquez D., Moncada-Habib T., Cassiano-Quezada F., Cadenhead K.S., Gómez-Martín D., De La Fuente-Sandoval C. (2023). Systemic Inflammation and Cortical Neurochemistry in Never-Medicated First Episode-Psychosis Individuals. Brain Behav. Immun..

[92] Parksepp M., Haring L., Kilk K., Taalberg E., Kangro R., Zilmer M., Vasar E. (2022). A Marked Low-Grade Inflammation and a Significant Deterioration in Metabolic Status in First-Episode Schizophrenia: A Five-Year Follow-Up Study. Metabolites.

[93] Chen L., Zheng W.-H., Du Y., Li X.-S., Yu Y., Wang H., Cheng Y. (2021). Altered Peripheral Immune Profiles in First-Episode, Drug-Free Patients With Schizophrenia: Response to Antipsychotic Medications. Front. Med..

[94] Shangguan F., Chen Z., Lv Y., Zhang X.-Y. (2023). Interaction between High Interleukin-2 and High Cortisol Levels Is Associated with Psychopathology in Patients with Chronic Schizophrenia. J. Psychiatr. Res..

[95] Wu Z.W., Yu H.H., Wang X., Guan H.Y., Xiu M.H., Zhang X.Y. (2021). Interrelationships Between Oxidative Stress, Cytokines, and Psychotic Symptoms and Executive Functions in Patients With Chronic Schizophrenia. Psychosom. Med..

[96] Ozdilli K., Mervan Aytac H., Ceren Tuncel F., Oyaci Y., Pehlivan M., Pehlivan S. (2024). Evaluation of Gene-Gene Interaction between the Interleukin (IL)-2 and IL-2RA Gene Polymorphisms in Schizophrenia Patients in the Turkish Population. Neurosci. J..

[97] Guo X., Kong L., Wen Y., Chen L., Hu S. (2024). Impact of Second-Generation Antipsychotics Monotherapy or Combined Therapy in Cytokine, Lymphocyte Subtype, and Thyroid Antibodies for Schizophrenia: A Retrospective Study. BMC Psychiatry.

[98] Dong Y., Zhu M., Li Y., Liu N., Wang X., Yang B., Li S., Li Z. (2024). Association of Cytokines Levels, Psychopathology and Cognition among CR-TRS Patients with Metabolic Syndrome. Schizophrenia.

[99] Lv M., Wang X., He X., Wang Z., Li X., Tan Y., Zhang X.Y. (2025). Obesity, Cytokines and Psychopathology in Patients with Chronic Schizophrenia. Front. Psychiatry.

[100] González-Blanco L., García-Portilla M.P., García-Álvarez L., De La Fuente-Tomás L., Iglesias García C., Sáiz P.A., Rodríguez-González S., Coto-Montes A., Bobes J. (2019). ¿Pueden ser la interleucina-2 y la interleucina-1β biomarcadores específicos de la sintomatología negativa en la esquizofrenia?. Rev. Psiquiatr. Salud Ment..

[101] Larsen J.B., Reitan S.K., Løberg E.-M., Rettenbacher M., Bruserud Ø., Larsen T.K., Anda L., Bartz-Johannessen C., Johnsen E., Kroken R.A. (2021). The Association between Cytokines and Psychomotor Speed in a Spectrum of Psychotic Disorders: A Longitudinal Study. Brain Behav. Immun. Health.

[102] Fenn-Moltu S., Deakin B., Drake R., Howes O.D., Lawrie S.M., Lewis S., Nikkheslat N., Walters J.T.R., MacCabe J.H., Mondelli V. (2023). The Association between Peripheral Inflammation, Brain Glutamate and Antipsychotic Response in Schizophrenia: Data from the STRATA Collaboration. Brain Behav. Immun..

[103] Clark D.N., Brown S.V., Xu L., Lee R.-L., Ragusa J.V., Xu Z., Milner J.D., Filiano A.J. (2025). Prolonged STAT1 Signaling in Neurons Causes Hyperactive Behavior. Brain Behav. Immun..

[104] Li X., Wu X., Li W., Yan Q., Zhou P., Xia Y., Yao W., Zhu F. (2023). HERV-W ENV Induces Innate Immune Activation and Neuronal Apoptosis via Linc01930/cGAS Axis in Recent-Onset Schizophrenia. Int. J. Mol. Sci..

[105] Ouyang L., Li D., Li Z., Ma X., Yuan L., Fan L., Yang Z., Zhang Z., Li C., He Y. (2022). IL-17 and TNF-β: Predictive Biomarkers for Transition to Psychosis in Ultra-High Risk Individuals. Front. Psychiatry.

[106] Murphy C.E., Walker A.K., O’Donnell M., Galletly C., Lloyd A.R., Liu D., Weickert C.S., Weickert T.W. (2022). Peripheral NF-κB Dysregulation in People with Schizophrenia Drives Inflammation: Putative Anti-Inflammatory Functions of NF-κB Kinases. Transl. Psychiatry.

[107] Hidese S. (2024). Search for Cerebrospinal Fluid Biomarkers in Patients with Major Psychiatric Disorders: Multiplex Immunoassay Findings and Proximity Extension Assay Prospects. Neuropsychopharmacol. Rep..

[108] Li M., Luo G., Qiu Y., Zhang X., Sun X., Li Y., Zhao Y., Sun W., Yang S., Li J. (2024). Negative Symptoms and Neurocognition in Drug-Naïve Schizophrenia: Moderating Role of Plasma Neutrophil Gelatinase-Associated Lipocalin (NGAL) and Interferon-Gamma (INF-γ). Eur. Arch. Psychiatry Clin. Neurosci..

[109] Sun X., Luo G., Li X., Wang J., Qiu Y., Li M., Li J. (2024). The Relationship between Inflammatory Markers, Clinical Characteristics, and Cognitive Performance in Drug-Naïve Patients with Schizophrenia. Eur. Arch. Psychiatry Clin. Neurosci..

[110] Miller B.J., Lemos H., Schooler N.R., Goff D.C., Kopelowicz A., Lauriello J., Manschreck T., Mendelowitz A., Miller D.D., Severe J.B. (2023). Longitudinal Study of Inflammation and Relapse in Schizophrenia. Schizophr. Res..

[111] He X., Ma Q., Fan Y., Zhao B., Wang W., Zhu F., Ma X., Zhou L. (2020). The Role of Cytokines in Predicting the Efficacy of Acute Stage Treatment in Patients with Schizophrenia. Neuropsychiatr. Dis. Treat..

[112] Jeppesen R., Borbye-Lorenzen N., Christensen R.H.B., Sørensen N.V., Köhler-Forsberg O., Skogstrand K., Benros M.E. (2024). Levels of Cytokines in the Cerebrospinal Fluid of Patients with Psychotic Disorders Compared to Individually Matched Healthy Controls. Brain Behav. Immun..

[113] Kosger F., Yigitaslan S., Essizoglu A., Gulec G., Dag Karatas R., Sevil Degirmenci S. (2020). Inflammation and Oxidative Stress in Deficit Schizophrenia. Arch. Neuropsychiatry.

[114] Corsi-Zuelli F., Quattrone D., Ragazzi T.C.C., Loureiro C.M., Shuhama R., Menezes P.R., Louzada-Junior P., Del-Ben C.M. (2024). Transdiagnostic Dimensions of Symptoms and Experiences Associated with Immune Proteins in the Continuity of Psychosis. Psychol. Med..

[115] Enache D., Nikkheslat N., Fathalla D., Morgan B.P., Lewis S., Drake R., Deakin B., Walters J., Lawrie S.M., Egerton A. (2021). Peripheral Immune Markers and Antipsychotic Non-Response in Psychosis. Schizophr. Res..

[116] Samoud S., Mtiraoui A., Zamali I., Galai Y., Hannachi N., Manoubi W., Nakhli J., Louzir H., Kissi Y.E. (2025). Comparative Analysis of Serum BAFF and IL-17 Levels Pre- and Post-Antipsychotic Treatment for Acute Schizophrenia. Int. J. Mol. Sci..

[117] Zhang Y., Shi H., Yang G., Yang Y., Li W., Song M., Shao M., Su X., Lv L. (2021). Associations between Expression of Indoleamine 2, 3-Dioxygenase Enzyme and Inflammatory Cytokines in Patients with First-Episode Drug-Naive Schizophrenia. Transl. Psychiatry.

[118] Chen D., Li H., Zhao Q., Song J., Lin C., Yu J. (2021). Effect of Risperidone Treatment on Insulin-like Growth Factor-1 and Interleukin-17 in Drug Naïve First-Episode Schizophrenia. Psychiatry Res..

[119] Yuan X., Yang Q., Yao Y., Song S., Zhou X., Liu H., Zhang K. (2024). Role of HOMA-IR and IL-6 as Screening Markers for the Metabolic Syndrome in Patients with Chronic Schizophrenia: A Psychiatric Hospital-Based Cross-Sectional Study. Eur. Arch. Psychiatry Clin. Neurosci..

[120] Skorobogatov K., De Picker L., Wu C.-L., Foiselle M., Richard J.-R., Boukouaci W., Bouassida J., Laukens K., Meysman P., Le Corvoisier P. (2024). Immune-Based Machine Learning Prediction of Diagnosis and Illness State in Schizophrenia and Bipolar Disorder. Brain Behav. Immun..

[121] Yeh T.-C., Chu H.-T., Tsai C.-K., Chang H.-A., Yang F.-C., Huang S.-Y., Liang C.-S. (2019). Distinct Inflammation Biomarkers in Healthy Individuals and Patients with Schizophrenia: A Reliability Testing of Multiplex Cytokine Immunoassay by Bland-Altman Analysis. Psychiatry Investig..

[122] Cyran A., Pawlak E., Piotrowski P., Bielawski T., Samochowiec J., Tyburski E., Chęć M., Rembacz K., Łaczmański Ł., Bieniek W. (2023). The Deficit Subtype of Schizophrenia Is Associated with a Pro-Inflammatory Phenotype but Not with Altered Levels of Zonulin: Findings from a Case-Control Study. Psychoneuroendocrinology.

[123] Chenniappan R., Nandeesha H., Kattimani S., Nanjaiah N.D. (2020). Interleukin-17 and Interleukin-10 Association with Disease Progression in Schizophrenia. Ann. Neurosci..

[124] Zheng Y., Cai X., Wang D., Chen X., Wang T., Xie Y., Li H., Wang T., He Y., Li J. (2024). Exploring the Relationship between Lipid Metabolism and Cognition in Individuals Living with Stable-Phase Schizophrenia: A Small Cross-Sectional Study Using Olink Proteomics Analysis. BMC Psychiatry.

[125] Borovcanin M.M., Minic Janicijevic S., Jovanovic I.P., Gajovic N.M., Jurisevic M.M., Arsenijevic N.N. (2020). Type 17 Immune Response Facilitates Progression of Inflammation and Correlates with Cognition in Stable Schizophrenia. Diagnostics.

[126] Feingold K.R., Feingold K.R., Ahmed S.F., Anawalt B., Blackman M.R., Boyce A., Chrousos G., Corpas E., de Herder W.W., Dhatariya K., Dungan K. (2000). Obesity and Dyslipidemia. Endotext.

[127] Nguyen J.C.D., Killcross A.S., Jenkins T.A. (2014). Obesity and Cognitive Decline: Role of Inflammation and Vascular Changes. Front. Neurosci..

[128] Zhilyaeva T.V., Rukavishnikov G.V., Manakova E.A., Mazo G.E. (2023). Serum Interleukin-6 in Schizophrenia: Associations with Clinical and Sociodemographic Characteristics. Consort. Psychiatr..

[129] Chen P., Yang H.-D., Wang J.-J., Zhu Z.-H., Zhao H.-M., Yin X.-Y., Cai Y., Zhu H.-L., Fu J.-L., Zhang X.-Z. (2024). Association of Serum Interleukin-6 with Negative Symptoms in Stable Early-Onset Schizophrenia. World J. Psychiatry.

[130] King S., Holleran L., Mothersill D., Patlola S., Rokita K., McManus R., Kenyon M., McDonald C., Hallahan B., Corvin A. (2021). Early Life Adversity, Functional Connectivity and Cognitive Performance in Schizophrenia: The Mediating Role of IL-6. Brain Behav. Immun..

[131] Gonzalez-Blanco L., Garcia-Portilla M.P., Dal Santo F., Garcia-Alvarez L., De La Fuente-Tomas L., Menendez-Miranda I., Bobes-Bascaran T., Saiz P.A., Bobes J. (2019). Predicting Real-World Functioning in Outpatients with Schizophrenia: Role of Inflammation and Psychopathology. Psychiatry Res..

[132] Ghasemi Noghabi P., Shahini N., Salimi Z., Ghorbani S., Bagheri Y., Derakhshanpour F. (2024). Elevated Serum IL-17 A and CCL20 Levels as Potential Biomarkers in Major Psychotic Disorders: A Case-Control Study. BMC Psychiatry.

[133] Guo T., Chen L., Luan L., Yang M., Zhang X., Yang H. (2024). Variations in Inflammatory Regulators in Male Patients with Chronic Schizophrenia Associated with Psychopathology and Cognitive Deficits. BMC Psychiatry.

[134] Maes M., Sirivichayakul S., Matsumoto A.K., Maes A., Michelin A.P., De Oliveira Semeão L., De Lima Pedrão J.V., Moreira E.G., Barbosa D.S., Geffard M. (2020). Increased Levels of Plasma Tumor Necrosis Factor-α Mediate Schizophrenia Symptom Dimensions and Neurocognitive Impairments and Are Inversely Associated with Natural IgM Directed to Malondialdehyde and Paraoxonase 1 Activity. Mol. Neurobiol..

[135] Şimşek Ş., Yıldırım V., Çim A., Kaya S. (2016). Serum IL-4 and IL-10 Levels Correlate with the Symptoms of the Drug-Naive Adolescents with First Episode, Early Onset Schizophrenia. J. Child Adolesc. Psychopharmacol..

[136] Fan Y., Gao Y., Ma Q., Zhao B., He X., Zhu F., Wang W., Ma X., Li Y. (2022). Grey Matter Volume and Its Association with Cognitive Impairment and Peripheral Cytokines in Excited Individuals with Schizophrenia. Brain Imaging Behav..

[137] Ntouros E., Karanikas E., Floros G., Andreou C., Tsoura A., Garyfallos G., Bozikas V.P. (2018). Social Cognition in the Course of Psychosis and Its Correlation with Biomarkers in a Male Cohort. Cognit. Neuropsychiatry.

[138] Ribeiro-Santos R., De Campos-Carli S.M., Ferretjans R., Teixeira-Carvalho A., Martins-Filho O.A., Teixeira A.L., Salgado J.V. (2020). The Association of Cognitive Performance and IL-6 Levels in Schizophrenia Is Influenced by Age and Antipsychotic Treatment. Nord. J. Psychiatry.

[139] Dahan S., Bragazzi N.L., Yogev A., Bar-Gad M., Barak V., Amital H., Amital D. (2018). The Relationship between Serum Cytokine Levels and Degree of Psychosis in Patients with Schizophrenia. Psychiatry Res..

[140] Malashenkova I.K., Ushakov V.L., Zakharova N.V., Krynskiy S.A., Ogurtsov D.P., Hailov N.A., Chekulaeva E.I., Ratushnyy A.Y., Kartashov S.I., Kostyuk G.P. (2021). Neuro-Immune Aspects of Schizophrenia with Severe Negative Symptoms: New Diagnostic Markers of Disease Phenotype. Sovrem. Tehnol. V Med..

[141] Xiu M.H., Yang G.G., Tan Y.L., Chen D.C., Tan S.P., Wang Z.R., Yang F.D., Okusaga O., Soares J.C., Zhang X.Y. (2014). Decreased Interleukin-10 Serum Levels in First-Episode Drug-Naïve Schizophrenia: Relationship to Psychopathology. Schizophr. Res..

[142] Pantovic-Stefanovic M., Velimirovic M., Jurisic V., Puric M., Gostiljac M., Dodic S., Minic I., Nesic M., Nikolic T., Petronijevic N. (2024). Exploring the Role of TNF-α, TGF-β, and IL-6 Serum Levels in Categorical and Noncategorical Models of Mood and Psychosis. Sci. Rep..

[143] Nani J.V., Almeida P.G.C., Noto C., Bressan R.A., Brietzke E., Hayashi M.A.F. (2022). Unraveiling the Correlation among Neurodevelopmental and Inflammatory Biomarkers in Patients with Chronic Schizophrenia. Nord. J. Psychiatry.

[144] Minic Janicijevic S., Jovanovic I.P., Gajovic N.M., Jurisevic M.M., Debnath M., Arsenijevic N.N., Borovcanin M.M. (2022). Galectin-3 Mediated Risk of Inflammation in Stable Schizophrenia, with Only Possible Secondary Consequences for Cognition. World J. Psychiatry.

[145] Schmitt Junior A.A., Primo De Carvalho Alves L., Padilha B.L., Da Rocha N.S. (2023). Serum Cytokine Variations among Inpatients with Major Depression, Bipolar Disorder, and Schizophrenia versus Healthy Controls: A Prospective ‘True-to-Life’ Study. Ther. Adv. Psychopharmacol..

[146] Carril Pardo C., Oyarce Merino K., Vera-Montecinos A. (2025). Neuroinflammatory Loop in Schizophrenia, Is There a Relationship with Symptoms or Cognition Decline?. Int. J. Mol. Sci..

[147] Liu J.-Y., Chen H.-Y., Lin J.-J., Lu M.-K., Tan H.-P., Jang F.-L., Lin S.-H. (2020). Alterations of Plasma Cytokine Biomarkers for Identifying Age at Onset of Schizophrenia with Neurological Soft Signs. Int. J. Med. Sci..

[148] Hoprekstad G.E., Kjelby E., Gjestad R., Fathian F., Larsen T.K., Reitan S.K., Rettenbacher M., Torsvik A., Skrede S., Johnsen E. (2023). Depression Trajectories and Cytokines in Schizophrenia Spectrum Disorders—A Longitudinal Observational Study. Schizophr. Res..

[149] Hernandez J.D., Li T., Ghannam H., Rau C.M., Masuda M.Y., Madura J.A., Jacobsen E.A., De Filippis E. (2024). Linking Adipose Tissue Eosinophils, IL-4, and Leptin in Human Obesity and Insulin Resistance. JCI Insight.

[150] Corsi-Zuelli F., Loureiro C.M., Shuhama R., Fachim H.A., Menezes P.R., Louzada-Junior P., Mondelli V., Del-Ben C.M. (2020). Cytokine Profile in First-Episode Psychosis, Unaffected Siblings and Community-Based Controls: The Effects of Familial Liability and Childhood Maltreatment. Psychol. Med..

[151] Michalczyk A., Tyburski E., Podwalski P., Waszczuk K., Rudkowski K., Kucharska-Mazur J., Mak M., Rek-Owodziń K., Plichta P., Bielecki M. (2022). Serum Inflammatory Markers and Their Associations with the Integrity of the Cingulum Bundle in Schizophrenia, from Prodromal Stages to Chronic Psychosis. J. Clin. Med..

[152] Arabska J., Wysokiński A., Brzezińska-Błaszczyk E., Kozłowska E. (2022). Serum Levels and in Vitro CX3CL1 (Fractalkine), CXCL8, and IL-10 Synthesis in Phytohemaglutinin-Stimulated and Non-Stimulated Peripheral Blood Mononuclear Cells in Subjects with Schizophrenia. Front. Psychiatry.

[153] Goldsmith D.R., Massa N., Pearce B.D., Wommack E.C., Alrohaibani A., Goel N., Cuthbert B., Fargotstein M., Felger J.C., Haroon E. (2020). Inflammatory Markers Are Associated with Psychomotor Slowing in Patients with Schizophrenia Compared to Healthy Controls. Npj Schizophr..

[154] Almulla A.F., Al-Rawi K.F., Maes M., Al-Hakeim H.K. (2021). In Schizophrenia, Immune-Inflammatory Pathways Are Strongly Associated with Depressive and Anxiety Symptoms, Which Are Part of a Latent Trait Which Comprises Neurocognitive Impairments and Schizophrenia Symptoms. J. Affect. Disord..

[155] Golimbet V., Lezheiko T., Mikhailova V., Korovaitseva G., Kolesina N., Plakunova V., Kostyuk G. (2022). A Study of the Association between Polymorphisms in the Genes for Interleukins IL-6 and IL-10 and Negative Symptoms Subdomains in Schizophrenia. Indian J. Psychiatry.

[156] Wang J., Xu H., Wang D., Wei G., Zhou H., Wang L., Zhou Y., Zhang X. (2021). The Interactive Effect of Genetic Polymorphisms of IL-10 and COMT on Cognitive Function in Schizophrenia. J. Psychiatr. Res..

[157] Năstase M.G., Vlaicu I., Trifu S.C. (2022). Genetic Polymorphism and Neuroanatomical Changes in Schizophrenia. Rom. J. Morphol. Embryol. Rev. Roum. Morphol. Embryol..

[158] Zakowicz P., Pawlak J., Kapelski P., Wiłkość-Dębczyńska M., Szałkowska A., Twarowska-Hauser J., Rybakowski J., Skibińska M. (2022). Genetic Association Study Reveals Impact of Interleukin 10 Polymorphisms on Cognitive Functions in Schizophrenia. Behav. Brain Res..

[159] Choi K.-Y., Choo J.M., Lee Y.-J., Lee Y., Cho C.-H., Kim S.-H., Lee H.-J. (2020). Association between the IL10 Rs1800896 Polymorphism and Tardive Dyskinesia in Schizophrenia. Psychiatry Investig..

[160] Lauridsen J.K., Olesen R.H., Vendelbo J., Hyde T.M., Kleinman J.E., Bibby B.M., Brock B., Rungby J., Larsen A. (2017). High BMI Levels Associate with Reduced mRNA Expression of IL10 and Increased mRNA Expression of iNOS (NOS_2_) in Human Frontal Cortex. Transl. Psychiatry.

[161] Fang X., Yu L., Wang D., Chen Y., Wang Y., Wu Z., Liu R., Ren J., Tang W., Zhang C. (2020). Association Between SIRT1, Cytokines, and Metabolic Syndrome in Schizophrenia Patients with Olanzapine or Clozapine Monotherapy. Front. Psychiatry.

[162] Pandey G.N., Rizavi H.S., Zhang H., Ren X. (2018). Abnormal Gene and Protein Expression of Inflammatory Cytokines in the Postmortem Brain of Schizophrenia Patients. Schizophr. Res..

[163] Yan J., Chen Y., Ju P., Gao J., Zhang L., Li J., Wang K., Zhang J., Li C., Xia Q. (2022). Network Association of Biochemical and Inflammatory Abnormalities with Psychiatric Symptoms in First-Episode Schizophrenia Patients. Front. Psychiatry.

[164] Noto M.N., Maes M., Nunes S.O.V., Ota V.K., Rossaneis A.C., Verri W.A., Cordeiro Q., Belangero S.I., Gadelha A., Bressan R.A. (2019). Activation of the Immune-Inflammatory Response System and the Compensatory Immune-Regulatory System in Antipsychotic Naive First Episode Psychosis. Eur. Neuropsychopharmacol..

[165] Amoli M.M., Khatami F., Arzaghi S.M., Enayati S., Nejatisafa A.-A. (2019). Over-Expression of TGF-Β1 Gene in Medication Free Schizophrenia. Psychoneuroendocrinology.

[166] Pan S., Zhou Y., Yan L., Xuan F., Tong J., Li Y., Huang J., Feng W., Chen S., Cui Y. (2022). TGF-Β1 Is Associated with Deficits in Cognition and Cerebral Cortical Thickness in First-Episode Schizophrenia. J. Psychiatry Neurosci..

[167] Kathuria A., Lopez-Lengowski K., Roffman J.L., Karmacharya R. (2022). Distinct Effects of Interleukin-6 and Interferon-γ on Differentiating Human Cortical Neurons. Brain Behav. Immun..

[168] Karampas A., Leontaritis G., Markozannes G., Asimakopoulos A., Archimandriti D.T., Spyrou P., Georgiou G., Plakoutsis M., Kotsis K., Voulgari P.V. (2025). Adiponectin, Resistin, Interleukin-4 and TGF-Β2 Levels in Treatment Resistant Schizophrenia Patients. Prog. Neuropsychopharmacol. Biol. Psychiatry.

[169] Sun Y., Zhu C., Huang L., Luo C., Ju P., Chen J. (2024). Identification of Key Modules in Metabolic Syndrome Induced by Second-Generation Antipsychotics Based on Co-Expression Network Analysis. Comput. Struct. Biotechnol. J..

[170] Bergin R., Kinlen D., Kedia-Mehta N., Hayes E., Cassidy F.C., Cody D., O’Shea D., Hogan A.E. (2022). Mucosal-Associated Invariant T Cells Are Associated with Insulin Resistance in Childhood Obesity, and Disrupt Insulin Signalling via IL-17. Diabetologia.

[171] Mangodt T.C., Van Herck M.A., Nullens S., Ramet J., De Dooy J.J., Jorens P.G., De Winter B.Y. (2015). The Role of Th17 and Treg Responses in the Pathogenesis of RSV Infection. Pediatr. Res..

[172] Matia-Garcia I., Vadillo E., Pelayo R., Muñoz-Valle J.F., García-Chagollán M., Loaeza-Loaeza J., Vences-Velázquez A., Salgado-Goytia L., García-Arellano S., Parra-Rojas I. (2021). Th1/Th2 Balance in Young Subjects: Relationship with Cytokine Levels and Metabolic Profile. J. Inflamm. Res..

[173] Penninx B.W.J.H., Lange S.M.M. (2018). Metabolic Syndrome in Psychiatric Patients: Overview, Mechanisms, and Implications. Dialogues Clin. Neurosci..

[174] Rarinca V., Vasile A., Visternicu M., Burlui V., Halitchi G., Ciobica A., Singeap A.-M., Dobrin R., Burlui E., Maftei L. (2024). Relevance of Diet in Schizophrenia: A Review Focusing on Prenatal Nutritional Deficiency, Obesity, Oxidative Stress and Inflammation. Front. Nutr..

[175] Ling Z., Cheng Y., Liu X., Xu X., Wu L., Shao L., Zhu Z., Ding W., Song Q., Zhao L. (2025). Schizophrenia-Associated Alterations in Fecal Mycobiota and Systemic Immune Dysfunction: A Cohort Study of Elderly Chinese Patients. Front. Immunol..

[176] Van Nimwegen L.J.M., Storosum J.G., Blumer R.M.E., Allick G., Venema H.W., De Haan L., Becker H., Van Amelsvoort T., Ackermans M.T., Fliers E. (2008). Hepatic Insulin Resistance in Antipsychotic Naive Schizophrenic Patients: Stable Isotope Studies of Glucose Metabolism. J. Clin. Endocrinol. Metab..

[177] Klemettilä J.-P., Kampman O., Seppälä N., Viikki M., Hämäläinen M., Moilanen E., Leinonen E. (2014). Cytokine and Adipokine Alterations in Patients with Schizophrenia Treated with Clozapine. Psychiatry Res..

[178] Chase K.A., Rosen C., Gin H., Bjorkquist O., Feiner B., Marvin R., Conrin S., Sharma R.P. (2015). Metabolic and Inflammatory Genes in Schizophrenia. Psychiatry Res..

[179] Soldevila-Matías P., Sánchez-Ortí J.V., Correa-Ghisays P., Balanzá-Martínez V., Selva-Vera G., Sanchis-Sanchis R., Iglesias-García N., Monfort-Pañego M., Tomás-Martínez P., Victor V.M. (2024). Exercise as a Promoter of Neurocognitive Improvement in People with Psychiatric Disorders and Comorbid Obesity: A Randomized Controlled Trial. Psychiatry Res..

[180] He J., Wei Y., Li J., Tang Y., Liu J., He Z., Zhou R., He X., Ren H., Liao Y. (2023). Sex Differences in the Association of Treatment-Resistant Schizophrenia and Serum Interleukin-6 Levels. BMC Psychiatry.

[181] Mednova I.A., Boiko A.S., Kornetova E.G., Parshukova D.A., Semke A.V., Bokhan N.A., Loonen A.J.M., Ivanova S.A. (2020). Adipocytokines and Metabolic Syndrome in Patients with Schizophrenia. Metabolites.

[182] Arabska J., Strzelecki D., Kozłowska E., Brzezińska-Błaszczyk E., Wysokiński A. (2020). The Association between Serum Levels of TNF-α and IL-6 in Schizophrenic Patients and Their Metabolic Status—A Case Control Study. J. Neuroimmunol..

[183] Bak M., Fransen A., Janssen J., Van Os J., Drukker M. (2014). Almost All Antipsychotics Result in Weight Gain: A Meta-Analysis. PLoS ONE.

[184] Barton B.B., Segger F., Fischer K., Obermeier M., Musil R. (2020). Update on Weight-Gain Caused by Antipsychotics: A Systematic Review and Meta-Analysis. Expert Opin. Drug Saf..

[185] Fountaine R.J., Taylor A.E., Mancuso J.P., Greenway F.L., Byerley L.O., Smith S.R., Most M.M., Fryburg D.A. (2010). Increased Food Intake and Energy Expenditure Following Administration of Olanzapine to Healthy Men. Obesity.

[186] Fehsel K. (2024). Metabolic Side Effects from Antipsychotic Treatment with Clozapine Linked to Aryl Hydrocarbon Receptor (AhR) Activation. Biomedicines.

[187] Liang J., Cai Y., Xue X., Li X., Li Z., Xu C., Xie G., Yu Y. (2022). Does Schizophrenia Itself Cause Obesity?. Front. Psychiatry.

[188] Grimm O., Kaiser S., Plichta M.M., Tobler P.N. (2017). Altered Reward Anticipation: Potential Explanation for Weight Gain in Schizophrenia?. Neurosci. Biobehav. Rev..

[189] Agarwal S.M., Stogios N., Ahsan Z.A., Lockwood J.T., Duncan M.J., Takeuchi H., Cohn T., Taylor V.H., Remington G., Faulkner G.E.J. (2022). Pharmacological Interventions for Prevention of Weight Gain in People with Schizophrenia. Cochrane Database Syst. Rev..

[190] Hong J., Bang M. (2020). Anti-Inflammatory Strategies for Schizophrenia: A Review of Evidence for Therapeutic Applications and Drug Repurposing. Clin. Psychopharmacol. Neurosci..

[191] Prabakaran S., Swatton J.E., Ryan M.M., Huffaker S.J., Huang J.-J., Griffin J.L., Wayland M., Freeman T., Dudbridge F., Lilley K.S. (2004). Mitochondrial Dysfunction in Schizophrenia: Evidence for Compromised Brain Metabolism and Oxidative Stress. Mol. Psychiatry.

[192] Laan W., Grobbee D.E., Selten J.-P., Heijnen C.J., Kahn R.S., Burger H. (2010). Adjuvant Aspirin Therapy Reduces Symptoms of Schizophrenia Spectrum Disorders: Results From a Randomized, Double-Blind, Placebo-Controlled Trial. J. Clin. Psychiatry.

[193] Attari A., Mojdeh A., Khalifeh Soltani F.A.S., Najarzadegan M.R. (2016). Aspirin Inclusion in Antipsychotic Treatment on Severity of Symptoms in Schizophrenia: A Randimized Clinical Trial. Iran. J. Psychiatry Behav. Sci..

[194] Schmidt L., Phelps E., Friedel J., Shokraneh F. (2019). Acetylsalicylic Acid (Aspirin) for Schizophrenia. Cochrane Database Syst. Rev..

[195] Weiser M., Zamora D., Levi L., Nastas I., Gonen I., Radu P., Matei V., Nacu A., Boronin L., Davidson M. (2021). Adjunctive Aspirin vs Placebo in Patients With Schizophrenia: Results of Two Randomized Controlled Trials. Schizophr. Bull..

[196] Cherneva D.I., Kehayova G., Dimitrova S., Dragomanova S. (2025). The Central Nervous System Modulatory Activities of N-Acetylcysteine: A Synthesis of Two Decades of Evidence. Curr. Issues Mol. Biol..

[197] Kerksick C., Willoughby D. (2005). The Antioxidant Role of Glutathione and N-Acetyl-Cysteine Supplements and Exercise-Induced Oxidative Stress. J. Int. Soc. Sports Nutr..

[198] Yolland C.O., Hanratty D., Neill E., Rossell S.L., Berk M., Dean O.M., Castle D.J., Tan E.J., Phillipou A., Harris A.W. (2020). Meta-Analysis of Randomised Controlled Trials with *N* -Acetylcysteine in the Treatment of Schizophrenia. Aust. N. Z. J. Psychiatry.

[199] Berk M., Copolov D., Dean O., Lu K., Jeavons S., Schapkaitz I., Anderson-Hunt M., Judd F., Katz F., Katz P. (2008). N-Acetyl Cysteine as a Glutathione Precursor for Schizophrenia—A Double-Blind, Randomized, Placebo-Controlled Trial. Biol. Psychiatry.

[200] Breier A., Liffick E., Hummer T.A., Vohs J.L., Yang Z., Mehdiyoun N.F., Visco A.C., Metzler E., Zhang Y., Francis M.M. (2018). Effects of 12-Month, Double-Blind N-Acetyl Cysteine on Symptoms, Cognition and Brain Morphology in Early Phase Schizophrenia Spectrum Disorders. Schizophr. Res..

[201] Akhondzadeh S., Tabatabaee M., Amini H., Ahmadiabhari S., Abbasi S., Behnam B. (2007). Celecoxib as Adjunctive Therapy in Schizophrenia: A Double-Blind, Randomized and Placebo-Controlled Trial. Schizophr. Res..

[202] Müller N., Krause D., Dehning S., Musil R., Schennach-Wolff R., Obermeier M., Möller H.-J., Klauss V., Schwarz M.J., Riedel M. (2010). Celecoxib Treatment in an Early Stage of Schizophrenia: Results of a Randomized, Double-Blind, Placebo-Controlled Trial of Celecoxib Augmentation of Amisulpride Treatment. Schizophr. Res..

[203] Rapaport M.H., Delrahim K.K., Bresee C.J., Maddux R.E., Ahmadpour O., Dolnak D. (2005). Celecoxib Augmentation of Continuously Ill Patients with Schizophrenia. Biol. Psychiatry.

[204] Wehring H.J., Elsobky T., McEvoy J.P., Vyas G., Richardson C.M., McMahon R.P., DiPaula B.A., Liu F., Sullivan K., Buchanan R.W. (2018). Adjunctive Minocycline in Clozapine-Treated Patients with Schizophrenia: Analyzing the Effects of Minocycline on Clozapine Plasma Levels. Psychiatr. Q..

[205] Möller M., Swanepoel T., Harvey B.H. (2015). Neurodevelopmental Animal Models Reveal the Convergent Role of Neurotransmitter Systems, Inflammation, and Oxidative Stress as Biomarkers of Schizophrenia: Implications for Novel Drug Development. ACS Chem. Neurosci..

[206] Panizzutti B., Skvarc D., Lin S., Croce S., Meehan A., Bortolasci C.C., Marx W., Walker A.J., Hasebe K., Kavanagh B.E. (2023). Minocycline as Treatment for Psychiatric and Neurological Conditions: A Systematic Review and Meta-Analysis. Int. J. Mol. Sci..

[207] Zhang L., Zheng H., Wu R., Zhu F., Kosten T.R., Zhang X.-Y., Zhao J. (2018). Minocycline Adjunctive Treatment to Risperidone for Negative Symptoms in Schizophrenia: Association with pro-Inflammatory Cytokine Levels. Prog. Neuropsychopharmacol. Biol. Psychiatry.

[208] Liu F., Zhang B., Xie L., Ruan Y., Xu X., Zeng Y., Messina L., Zhao J., Fan X. (2018). Changes in Plasma Levels of Nitric Oxide Metabolites and Negative Symptoms after 16-Week Minocycline Treatment in Patients with Schizophrenia. Schizophr. Res..

[209] Girgis R.R., Ciarleglio A., Choo T., Haynes G., Bathon J.M., Cremers S., Kantrowitz J.T., Lieberman J.A., Brown A.S. (2018). A Randomized, Double-Blind, Placebo-Controlled Clinical Trial of Tocilizumab, an Interleukin-6 Receptor Antibody, for Residual Symptoms in Schizophrenia. Neuropsychopharmacology.

[210] Miller B.J., Dias J.K., Lemos H.P., Buckley P.F. (2016). An Open-Label, Pilot Trial of Adjunctive Tocilizumab in Schizophrenia. J. Clin. Psychiatry.

[211] Strube W., Aksar A., Bauer I., Barbosa S., Benros M., Blankenstein C., Campana M., Davidovic L., Glaichenhaus N., Falkai P. (2023). Effects of Add-on Celecoxib Treatment on Patients with Schizophrenia Spectrum Disorders and Inflammatory Cytokine Profile Trial (TargetFlame): Study Design and Methodology of a Multicentre Randomized, Placebo-Controlled Trial. J. Neural Transm..

[212] Jeppesen R., Christensen R.H.B., Pedersen E.M.J., Nordentoft M., Hjorthøj C., Köhler-Forsberg O., Benros M.E. (2020). Efficacy and Safety of Anti-Inflammatory Agents in Treatment of Psychotic Disorders–A Comprehensive Systematic Review and Meta-Analysis. Brain Behav. Immun..

